# Machine-learning-based architecting of magnetoresistance phase diagrams in anomalous Hall systems

**DOI:** 10.1093/nsr/nwag228

**Published:** 2026-04-20

**Authors:** Ganyu Chen, Xiangyu Bi, Zeya Li, Xiao Feng, Yang Feng, Feng Qin, Junwei Huang, Ling Zhou, Ke He, Toshiya Ideue, Qi-Kun Xue, Hongtao Yuan

**Affiliations:** National Laboratory of Solid State Microstructures, College of Engineering and Applied Sciences, Collaborative Innovation Center of Advanced Microstructures, Nanjing University, Nanjing 210093, China; National Laboratory of Solid State Microstructures, College of Engineering and Applied Sciences, Collaborative Innovation Center of Advanced Microstructures, Nanjing University, Nanjing 210093, China; Quantum Science Center of Guangdong-Hong Kong-Macao Greater Bay Area, Shenzhen 518045, China; State Key Laboratory of Low Dimensional Quantum Physics and Department of Physics, Tsinghua University, Beijing 100084, China; Beijing Academy of Quantum Information Sciences, Beijing 100193, China; National Laboratory of Solid State Microstructures, College of Engineering and Applied Sciences, Collaborative Innovation Center of Advanced Microstructures, Nanjing University, Nanjing 210093, China; National Laboratory of Solid State Microstructures, College of Engineering and Applied Sciences, Collaborative Innovation Center of Advanced Microstructures, Nanjing University, Nanjing 210093, China; Quantum Science Center of Guangdong-Hong Kong-Macao Greater Bay Area, Shenzhen 518045, China; State Key Laboratory of Low Dimensional Quantum Physics and Department of Physics, Tsinghua University, Beijing 100084, China; Quantum Phase Electronic Center and Department of Applied Physics, The University of Tokyo, Tokyo 113-8654, Japan; Quantum Science Center of Guangdong-Hong Kong-Macao Greater Bay Area, Shenzhen 518045, China; State Key Laboratory of Low Dimensional Quantum Physics and Department of Physics, Tsinghua University, Beijing 100084, China; Department of Physics and Guangdong Basic Research Center of Excellence for Quantum Science, Southern University of Science and Technology, Shenzhen 518055, China; National Laboratory of Solid State Microstructures, College of Engineering and Applied Sciences, Collaborative Innovation Center of Advanced Microstructures, Nanjing University, Nanjing 210093, China; Jiangsu Physical Science Research Center, Nanjing 210093, China

**Keywords:** anomalous Hall effect, magnetoresistance, machine learning, ferromagnetism, two-band model

## Abstract

The nontrivial magnetoresistance in anomalous Hall systems (AH-MR) plays a crucial role in understanding electron dynamics in condensed matter systems. Unlike conventional Hall resistance reflecting the cyclotron motion of electrons under magnetic fields, anomalous Hall magnetoresistance typically stems from Berry-curvature-induced anomalous velocity or electron scattering events in anomalous Hall systems. Therefore, multiple parameters—such as carrier density, electrical conductivity and anomalous Hall conductivity—offer the capability for tailoring AH-MR associated phenomena including quantum anomalous Hall effect, colossal magnetoresistance and topological phase transition. However, the high-dimensional nature of these parameters hinders the global understanding of AH-MR and related electronic transport behavior. Here we employ machine learning algorithms to architect AH-MR phase diagrams by analyzing over 2000 000 AH-MR curves generated from a two-band model with five adjustable parameters. We found these curves can be clustered into 13 distinct AH-MR states using the mean-shift algorithm and established topological networks to describe transitions between them, offering designing transition paths to switch AH-MR states by tuning selected electronic parameters. Our experimental AH-MR results on gated Fe_5_GeTe_2_ nanoflakes—serving as a validation dataset—verify the reliability of the obtained topological relationships and landscape phase diagrams. Such a machine-learning-assisted approach of high-dimensional data processing offers a powerful methodology for investigating spin-dependent transport phenomena.

## Teaser

This article demonstrates machine-learning-aided global mapping of all accessible magnetoresistance curves to construct five-parameter-dimension phase diagrams, heatmap, and topological networks of magnetoresistance transitions for understanding the complex anomalous Hall systems.

## INTRODUCTION

Anomalous Hall effect and resulting nontrivial magnetoresistance play a pivotal role in understanding the interplay between spin-orbit coupling, Berry curvature and electronic band structures in condensed matter [1–5]. According to the Drude theory describing the motion of electrons, the mobile carriers under an external magnetic field would experience certain deflection due to the Lorentz force, typically producing parabolic magnetoresistance at low fields and saturation behavior at high fields [6]. In contrast, magnetoresistance in anomalous Hall systems (AH-MR) exhibits rich hysteretic behavior arising from band topology, side jump or skew scattering on electron motion [1,7], and thus, the distinct magnetoresistance characteristics such as the double-peak hysteresis in quantum anomalous Hall effect (QAHE) [8–13], the nonsaturating resistance in giant magnetoresistance (GMR) [14–16] or the central peak hysteresis in the spin Hall effect [17–19] emerge and can be effectively modulated by the electronic parameters including carrier density, electrical conductivity and anomalous Hall conductivity. In particular for those multiband anomalous Hall systems with multiple occupied energy bands around the Fermi level, the high dimensionality nature of those tunable electronic parameters can always introduce the inevitable complexity in interpreting AH-MR curves. Therefore, architecting a global phase diagram to unravel the intrinsic relationships between AH-MR characteristics and the electronic parameters is of great significance, yet remains elusive.

In this article, we demonstrate a machine-learning-aided landscape mapping of AH-MR characteristics and architect the global phase diagrams of all these AH-MR states with over 2000 000 AH-MR curves generated using the two-band model with five electronic parameters for describing magnetotransport. Employing the unsupervised machine-learning mean-shift algorithm, we found all these AH-MR curves can be clustered into 13 distinct AH-MR types based on such a massive dataset. Through analyzing an extensive AH-MR dataset using a trained self-normalizing neural network, we generated a quantitative heatmap to reveal the transition probabilities between AH-MR states, and further developed topological relationship networks to map the role of electronic parameters on the pairwise AH-MR transitions, offering a universal framework to design AH-MR transitions via tuning specific electronic parameters. Remarkably, experimental AH-MR data from gated Fe_5_GeTe_2_ nanoflakes, serving as a validation dataset, well verify certain transitions within our phase diagrams and topological networks. Architecting such phase diagrams can help predict the GMR and quantum anomalous Hall states, establishing a comprehensive strategy to realize AH-MR-associated quantum phenomena and spintronic devices.

## RESULTS

### Machine-learning-assisted classification for AH-MR states

**Figure 1.**
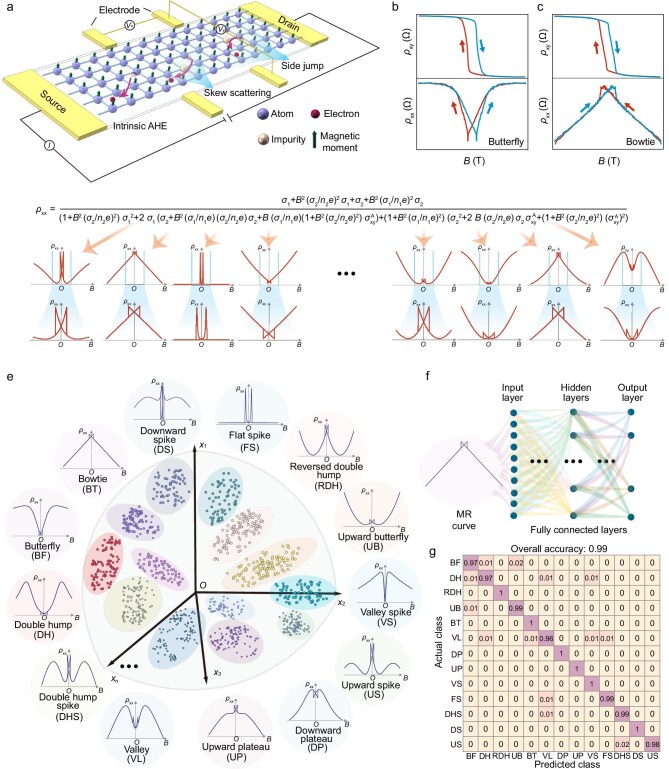
AH-MR generated using the two-band model. (a) Schematic of the electronic transport in anomalous Hall systems. A voltage is applied to the system, and the resulting current *I*, Hall voltage ${V}_{\rm y}$, and longitudinal voltage ${V}_{\rm x}$ are measured. The Hall resistance ${R}_{\rm xy}$ and the longitudinal resistance ${R}_{\rm xx}\ $can be obtained from ${R}_{\rm xy} = \frac{{{V}_{\rm y}}}{I}$ and ${R}_{\rm xx} = \frac{{{V}_{\rm x}}}{I}$. (b and c) Hall resistance and MR obtained from Fe_5_GeTe_2_ at $T = 50$ K, ${V}_{\mathrm{g}} = 2.5\ $V and at $T = 150$ K, ${V}_{\mathrm{g}} = 1.9\ $V, respectively. (d) AH-MR expression based on the two-band model, along with a variety of MR curves generated by this model. (e) Schematic of AH-MR curve clustering based on density-based clustering methods. Each AH-MR curve is discretized into 531 points and can thus be represented by a point in a 531-dimensional space (see Methods and Note S3). Different clusters are illustrated by ellipses of different colors. Thirteen unique AH-MR state types, as indicated by the colored ellipses, were obtained. AH-MR types and their abbreviations given are as follows: bowtie (BT), flat-spike (FS), butterfly (BF), upward-butterfly (UB), valley (VL), upward-plateau (UP), double-hump (DH), valley-spike (VS), downward-spike (DS), reversed-double-hump (RDH), double-hump-spike (DHS), downward-plateau (DP), upward-spike (US). (f) Schematic of AH-MR type recognition model trained using the neural network algorithm. (g) Normalized confusion matrix of the trained neural network.

We intentionally chose the anomalous Hall system for machine-learning-aided magnetoresistance analysis because of the following three reasons. First, the anomalous Hall effect arises from anomalous velocity [7,20,21], skew scattering and side jump [22–24] (Fig. 1a), and hosts rich interactions among magnetism, spin-orbit coupling and Berry curvature. These abundant interactions bring about complex magnetoresistance behavior, thus providing a practical platform to explore magnetoresistance phenomena and the profound physics therein. Second, since the magnetoresistance in anomalous Hall systems can be well described by the two-band model [25,26] with five electronic parameters (electrical conductivities of two carriers ${\sigma }_1$ and ${\sigma }_2$, their carrier densities ${n}_1$ and ${n}_2$ and the anomalous Hall conductivity $\sigma _{xy}^{\mathrm{A}}$), such a high-dimensional parameter nature is challenging for understanding AH-MR in a quantitative way, but it can serve as an excellent platform for demonstrating how machine learning solves problems in high-dimensional multi-parameter systems. Third, in certain anomalous Hall systems where the Hall resistance ${\rho }_{xy}( B )$ exhibits similar shapes while the AH-MR can manifest significantly different characteristics (Fig. 1b and c), implying that exploring AH-MR in such a scenario can always offer deep insights for the electronic scattering events associated with Berry curvature, skew scattering or side jump. Based on the above three reasons, we chose the anomalous Hall system for machine-learning-aided magnetoresistance analysis.

To explore all feasible AH-MR states, we used 2274 700 sets of AH-MR curves as the dataset for clustering AH-MR curves based on unsupervised machine learning algorithms. Figure 1d illustrates the generation process for AH-MR curves from the two-band model, with ${\sigma }_1$, ${\sigma }_2$ and $\sigma _{xy}^{\mathrm{A}}$ ranging from 10^−5^ S to 1 S, ${n}_1$ and ${n}_2$ ranging from 10^12^ to 10^15^ cm^−2^, which is a typical parameter range for ferromagnetic materials [27,28]. Note that by applying density-based clustering methods (details in Figs S1 and S2), all these 2274 700 sets of MR data can be categorized into only 13 types of AH-MR states, as shown in Fig. 1e. These curves have been widely observed in experiments. For example, the bowtie-type AH-MR is characterized by a nearly linear increase in magnetoresistance with decreasing magnetic field strength, accompanied by an abrupt resistance change at the coercive fields. Such behavior has been frequently observed in materials including Fe_5_GeTe_2_, CrTe_2_ and others [29–31]. Remarkably, the four most prevalent AH-MR curves—*bowtie* (29.09%), *flat-spike* (20.31%), *butterfly* (19.65%) and *upward-butterfly* (12.95%)—collectively represent 82% of all cases (details in Fig. S3 and Table S1), underscoring their significance in AH-MR studies. Such a data-driven AH-MR categorization simplifies the magnetoresistance research in condensed matter physics and spintronic devices.

We trained a self-normalizing neural network (SNN) as a classification tool that utilizes a vector of ${\rho }_{xx}$ values as the data input and generates the AH-MR type as the output result (details for SNN given in Fig. S4). Figure 1f shows the SNN architecture with one input layer, eight hidden layers and one output layer. For SNN training, we assembled a comprehensive dataset containing ∼110 representative curves per AH-MR type, with an 80:20 split between training and validation data. Note that our trained model achieved exceptional accuracy, reaching 0.99 on the training data and 0.97 on the validation dataset, with an overall accuracy of 0.99 across both datasets, as demonstrated by the normalized confusion matrices in Figs 1g and S5. This high-accuracy SNN model provides a powerful tool for categorizing all feasible AH-MR curves and for architecting landscape phase diagrams of AH-MR states.

### Architecting global phase diagrams of AH-MR states

**Figure 2.**
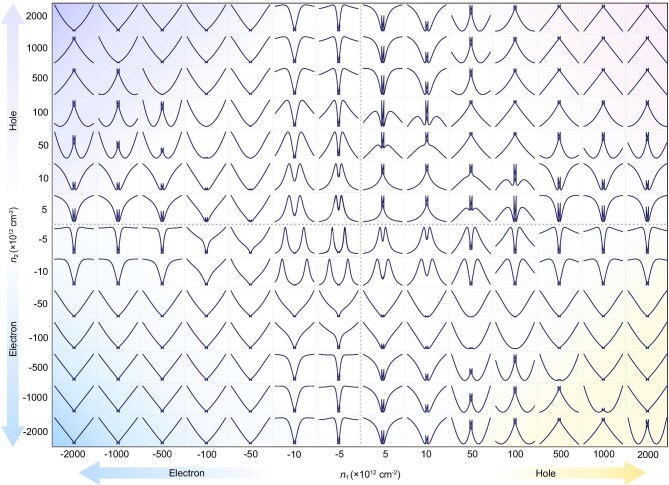
AH-MR curves obtained from the two-band model. The electrical conductivities of the two carriers are set to ${\sigma }_1 = 0.100\ $S and ${\sigma }_2 = $ 0.095 S, while the anomalous Hall conductivity is fixed at $\sigma _{\rm xy}^{\mathrm{A}}$  $=$ 0.050 S. The bottom *x*-axis corresponds to the carrier density of the first carrier, and the left y-axis represents that of the second carrier. Each plot displays the magnetoresistance curve obtained from the two-band model under the given parameters. A negative carrier density indicates an electron-type carrier, whereas a positive carrier density denotes a hole-type carrier. Different AH-MR curves can be achieved and further evolve to each other by systematically changing ${n}_1$, ${n}_2$.

**Figure 3.**
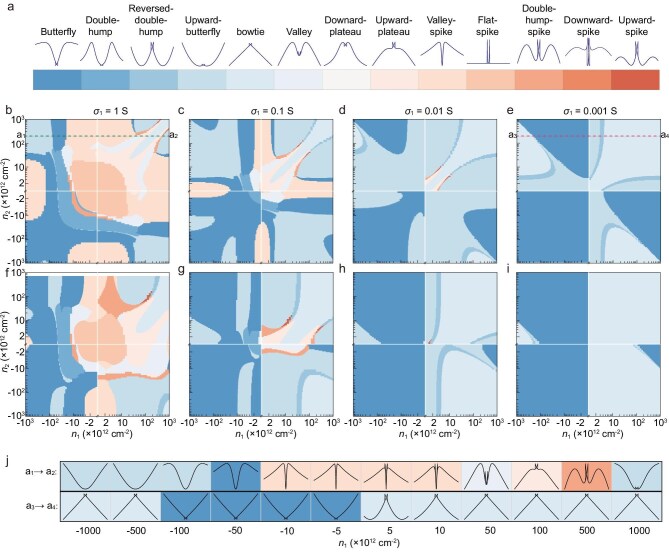
Phase diagrams illustrating the AH-MR type as a function of carrier densities. (a) Color bar showing the color representation of different AH-MR types. (b–i) Phase diagrams of AH-MR type with varying carrier densities, where ${\sigma }_2 = 0.95\ {\sigma }_1$. From b to e, $\ {\sigma }_1$ equals to 1 S, 0.1 S, 0.01 S and 0.001 S, respectively. In each diagram, $\sigma _{\rm xy}^{\mathrm{A}}$ remains at 0.08$\ {\sigma }_1$. (f–i) Phase diagrams of AH-MR types with varying carrier densities, where ${\sigma }_2 = 0.1\ {\sigma }_1$. From f to i,$\ \ {\sigma }_1$ equals to 1 S, 0.1 S, 0.01 S and 0.001 S, respectively. In each diagram, $\sigma _{\rm xy}^{\mathrm{A}}$ remains at 0.08$\ {\sigma }_1$. (j) MR curves evolutions along the dashed lines a_1_ → a_2_ and a_3_→ a_4_ in b and d. Both lines correspond to ${n}_2 = 2 \times {10}^{14}$ cm^−2^.

SNN can help us experience all possible AH-MR curves in the high-dimensional parameter space in the two-band model. Figures 2 and 3a–i show the AH-MR phase diagrams as a function of ${n}_1$ and ${n}_2$ with fixed ${\sigma }_1$, ${\sigma }_2$ and $\sigma _{xy}^{\mathrm{A}}$ values (Figs S6–S8 for those phase diagrams as a function of electrical conductivities). Such phase diagrams provide deep insights to understand the transition paths among AH-MR states and the underlying emergent magnetoresistance phenomena when we change those electronic parameters. For example, the linecut evolution of AH-MR curves along the green dashed line shown in Fig. 3b demonstrates that, as ${n}_1$ increases, AH-MR features transit sequentially from *upward-butterfly* to *butterfly, valley-spike, valley, upward-plateau, double-hump-spike*, and finally back to *upward-butterfly* (Fig. 3j). While for the red dashed line in Fig. 3e, AH-MR features transit from *bowtie* to *butterfly* and finally returning to *bowtie*. These two paths represent typical AH-MR evolutions in high- and low-conductivity regimes (see Figs S9–12 for more linecut evolutions and Fig. S13 for AH-MR phase diagrams with negative $\sigma _{xy}^{\mathrm{A}}$ values). Similarly, the phase diagrams with different conductivity ${\sigma }_1$ (Fig. 3b–i) can reveal the critical role of electrical conductivity in designing the AH-MR features. When ${\sigma }_1$ and ${\sigma }_2$ are comparable (e.g. ${\sigma }_2 = 0.95\ {\sigma }_1$; Fig. 3b–e), both carriers contribute equally to the AH-MR features, while when ${\sigma }_1$ is much larger than ${\sigma }_2$ (e.g. ${\sigma }_1 = 0.1 {\sigma }_2$; Fig. 3f–i), the AH-MR type varies more sharply along ${n}_1$ direction than along ${n}_2$ direction, indicating that higher conductivity can drive AH-MR transitions in a more effective way. These phase diagrams clearly illustrate the tuning strategies for desired AH-MR features through modulating electronic parameters. Under such a multi-parameter-controlled scenario, machine learning can more effectively analyze and resolve such complex situations.

### Topological relationship networks for AH-MR states

**Figure 4.**
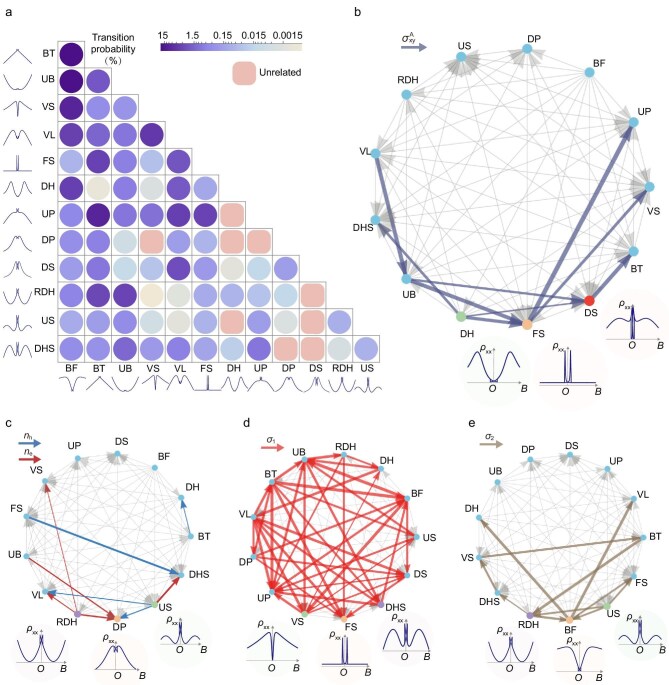
Relationships between AH-MR state types and electronic transport parameters. (a) Heatmap illustrating the connections between different AH-MR types. A colored circle represents the proportion of transitions between the corresponding two state types out of all recorded changes, and a light orange rectangle indicates the absence of transitions between the corresponding two types. (b–e) Topological networks depicting how electronic transport parameter changes drive AH-MR state transitions. Light blue vertices represent AH-MR types, and lines with arrows indicate type transitions. Thicker lines indicate a higher percentage of transitions between the corresponding types. In each panel, the dominant tuning parameter is represented by a colored directed line, with the direction indicating an increase in the value of the parameter. For instance, in the BT-to-DS transition, the dominant parameter change is an increase in $\sigma _{\rm xy}^{\mathrm{A}}$. b highlights transitions primarily governed by changes in anomalous Hall conductivity ($\sigma _{\rm xy}^{\mathrm{A}}$), with other transitions shown in gray. An increase in $\sigma _{\rm xy}^{\mathrm{A}}$ refers to a rise in magnitude (more positive or less negative). (c) Highlights transitions dominated by carrier density changes, where ${n}_{\mathrm{e}}$ and ${n}_{\mathrm{h}}$ denote electron and hole densities, respectively. (d and e) highlight transitions dominated by electrical conductivity changes. ${\sigma }_1$ and ${\sigma }_2\ $represent the conductivities of the majority and minority carriers, respectively.

Figure 4a presents a heatmap illustrating the transition probabilities (denoted as correlation coefficients here) between different AH-MR. Here, the correlation coefficient is defined by the ratio of the number of observed transitions between two AH-MR states to the total number of recorded transitions (see Methods and Note S6). One can see, from a total of 78 pairwise connections among 13 AH-MR states, that the transition between *bowtie* and *butterfly* AH-MR is the most common one, with a correlation coefficient of over 15% (suggesting strong connection between *bowtie* and *butterfly* curves). In contrast, transitions involving *downward-spike* and *upward-spike* AH-MR are rare, with ignorable correlation coefficients typically below 0.1% (suggesting that spike-featured states exhibit weak connections with others). Most correlation coefficients fall within the 1%–10% range. Notably, *bowtie* and *butterfly* AH-MR are indeed frequently observed transiting between each other in magnetic materials such as Fe_5_GeTe_2_ [29,30], Co_3_Sn_2_S_2_ [32,33] and CrTe_2_ [31] upon changing temperature, gate voltage or sample thickness—consistent with our findings, as their electronic parameters fall within a similar range as discussed in this study.

Figure 4b–e present the topological relationship networks between these 13 types of AH-MR states, revealing how important anomalous Hall conductivity $\sigma _{xy}^{\mathrm{A}}$, hole density ${n}_{\mathrm{h}}$, electron density ${n}_{\mathrm{e}}$, electrical conductivities ${\sigma }_1$ and ${\sigma }_2$ are to the realization of each pairwise AH-MR transition. One can see in Fig. 4b that tuning $\sigma _{xy}^{\mathrm{A}}$ can effectively induce transitions among the *downward-spike, flat-spike, double-hump* and *upward-butterfly* types, in which the directed lines represent such transitions with the arrow denoting the direction of parameter increase and the thickness indicating the magnitude of the correlation coefficient (thicker lines denote higher coefficients). By comparing the number of directed lines in the topological relationship network figures (Fig. 4b–e, reflecting the role of $\sigma _{xy}^{\mathrm{A}}$, ${n}_{\mathrm{h}}$, ${n}_{\mathrm{e}}$, ${\sigma }_1$ and$\ {\sigma }_2$), one can see that more transitions are driven by changing conductivity ${\sigma }_1$ of the majority carrier, suggesting that ${\sigma }_1$ plays a more significant role in the AH-MR behavior than other parameters. Taking *valley-spike* AH-MR curve as an example, nine AH-MR transitions toward it can be realized by tuning ${\sigma }_1$, while significantly fewer transitions occur with other parameters: only two by tuning ${\sigma }_2$, just one by tuning $\sigma _{xy}^{\mathrm{A}}$ or$\ {n}_{\mathrm{e}}$ and none by tuning ${n}_{\mathrm{h}}$. Such network topology relationships reveal the complex parameter influence on these AH-MR transitions, offering deep insights for a rational design of AH-MR behaviors and shedding light on the underlying mechanisms, including Berry curvature, skew scattering and side jump.

### Experimental verification of AH-MR phase diagrams

**Figure 5.**
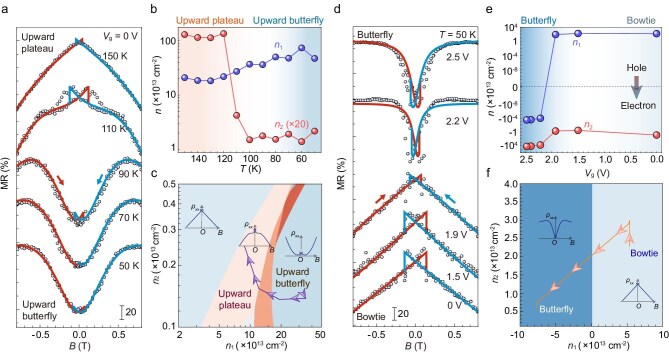
AH-MR transitions observed in Fe_5_GeTe_2_ flakes. (a) Temperature-dependent magnetoresistance of Fe_5_GeTe_2_ from $50\ $K to $150\ $K at ${V}_{\mathrm{g}} = 0$. White points denote experimental data, while red and blue curves represent fitted results. (b) Carrier densities as a function of temperature obtained from Fe_5_GeTe_2_ at ${V}_{\mathrm{g}} = 0$. Light orange and light blue regions indicate *bowtie*- and *butterfly*-type AH-MR, respectively. (c) Phase diagram illustrating AH-MR type evolution with carrier densities at ${\sigma }_1 = 0.1\ $S, ${\sigma }_2 = 0.01$ S and $\sigma _{\rm xy}^{\mathrm{A}} = 0.008$ S. The AH-MR type evolution shown in (b) roughly follows the path in shown in deep purple. The color representation of each AH-MR type is the same as those in Fig. 3. The insets show the corresponding AH-MR curves. Note that the path is an illustration of the evolution trend, and the AH-MR type evolution in b does not follow this path exactly. (d) Gate-dependent magnetoresistance data of Fe_5_GeTe_2_ from ${V}_{\mathrm{g}} = 0$ to $2.5\ $V at $T = 50\ $K, respectively. (e) Carrier densities of Fe_5_GeTe_2_ at 50 K as a function of gate voltage. Light blue and dark blue regions correspond to *bowtie*- and *butterfly*-type AH-MR, respectively. (f) Phase diagram illustrating AH-MR type evolution with carrier densities at ${\sigma }_1 = 1$ mS, ${\sigma }_2 = 0.1$ mS and $\sigma _{\rm xy}^{\mathrm{A}} = 0.08$ mS. The AH-MR type evolution shown in (e) roughly follows the orange path.

To experimentally confirm the validation of the obtained phase diagrams, we performed anomalous Hall and magnetotransport measurements to extract electronic parameters by curve fitting analyses. It should be addressed that changing temperature or gate voltage can serve as a powerful way to modify the five electronic parameters in the two-band model and tune the shape of AH-MR curves. Here the ferromagnetic material Fe_5_GeTe_2_ was selected as the AH-MR platform because of its rich magnetic properties [29,34] and tunable carrier density [29,35]. Figure 5a presents the magnetoresistance data and corresponding fitted curves for a Fe_5_GeTe_2_ Hall bar device under different temperatures. The excellent agreement between the experimental data and fitted curves enables reliable extraction of electronic parameters and identification of AH-MR states (see Figs S14–S21). One can see an AH-MR transition at ∼110 K from *upward-butterfly* type at lower temperatures to the *upward-plateau* type at higher temperatures, while the carrier density of minority carriers sharply increases also at ∼110 K with warming temperature (Fig. 5b), suggesting that such a transition in AH-MR type is driven by the carrier density increase (minority carriers). Note that such an experimental observation can be well described within the framework of our phase diagram (purple path shown in Fig. 5c), verifying that a carrier density increase for minority carriers indeed drives the transition from *upward-butterfly* to *upward-plateau* AH-MR state.

In order to further confirm the validation of our phase diagrams, we performed gating experiments on Fe_5_GeTe_2_ Hall bar devices and fitted the obtained magnetoresistance data at different gate voltages. One can see in Fig. 5d that the AH-MR curves change the feature from *bowtie* (${V}_{\mathrm{g}}$ < 1.9 V) to *butterfly* (${V}_{\mathrm{g}}$ > 1.9 V) with increased gate voltage, while the carrier changes from hole to electron at the same voltage (${V}_{\mathrm{g}}$ = 1.9 V) during increasing gate voltage (Fig. 5e). The simultaneous change in AH-MR feature (*bowtie* to *butterfly*) and carrier type (hole to electron) indicates that the AH-MR transition is driven by the carrier type change (majority carriers). Note that such experimental results can also be well described within our phase diagram (orange path in Fig. 5f), verifying that the change of majority carrier type from the hole to the electron indeed drives the transition from the *bowtie* to *butterfly* AH-MR states. Interestingly, considering that Fe_5_GeTe_2_ usually exhibits remarkable magnetic anisotropy transitions by gating or temperature cooling [29], the observed AH-MR transition here suggests that the evolution of AH-MR feature may also serve as one unique strategy to reflect magnetic anisotropy transitions and the electronic parameter evolution in a quantitative way. The excellent agreement between these experimental observations and our machine-learning-assisted phase diagrams strongly validates the reliability of the obtained phase diagrams and our analytical strategy.

To demonstrate prospective applications of the machine-learning-assisted AH-MR analysis, we further explore large magnetoresistance (LMR) and QAHE with this approach. LMR is characterized by a significant increase in magnetoresistance (MR) under external magnetic fields, including phenomena such as GMR, colossal magnetoresistance and nonsaturating magnetoresistance. Interestingly, we notice that the parameter region conducive to LMR can be effectively identified by analyzing AH-MR phase diagrams. Figure S22 presents the phase diagram of ${r}_{{\mathrm{LMR}}}$ (defined by ${r}_{{\mathrm{LMR}}} = \frac{{{\rho }_{xx}( {16\ {\mathrm{T}}} ) - {\rho }_{xx}( 0 )}}{{{\rho }_{xx}( 0 )}}$) as a function of carrier density, highlighting the parameter region to realize LMR phenomena in ferromagnetic materials with $\sigma _{xy}^{\mathrm{A}}\not= 0$. One can see in Figs S22a and S23 that the LMR phenomena mainly occur when electron density is higher than hole density, showing a bow-shaped region in red color that extends across the second, third and fourth quadrants of LMR phase diagrams. The total area of the bow-shaped region takes up less than 5% within a typical LMR phase diagram, implying it is really difficult to experimentally find this particular area with LMR phenomena while this area can be identified effectively through analyzing the AH-MR phase diagrams. Remarkably, even for a nonmagnetic material with $\sigma _{xy}^{\mathrm{A}} = 0$ (Fig. S22c and d), the ${r}_{{\mathrm{LMR}}}$ values exceeding 20 000% can be achieved in those scenarios with balanced electron-hole densities, which has been experimentally demonstrated by the nonsaturating MR reported in WTe_2_ [36]. On the other hand, since the MR value at the coercive field ($\rho _{xx}^{{H}_{\mathrm{C}}})$ in QAHE can always reflect the degree of magnetic disorder in the material [37], to study $\rho _{xx}^{{H}_{\mathrm{C}}}$ is of great significance for understanding and tailoring the ground states of QAHE. Figure S24 presents a phase diagram of $\rho _{xx}^{{H}_{\mathrm{C}}}$ as a function of carrier density in anomalous Hall systems. One can see that lower carrier density (higher mobility, since $\mu = \frac{\sigma }{{ne}}$) promotes the quantization of $\rho _{xx}^{{H}_{\mathrm{C}}}$, illustrating the same trend as the reported observation that the $\rho _{xx}^{{H}_{\mathrm{C}}}$ approaches the quantized value of *h*/*e*^2^ as the carrier mobility increases in magnetic topological insulators with QAHE phenomena [37–39]. Note that although we cannot obtain quantized states using the two-band model, this method can help us find the evolution path from nonquantized states to quantized states. These successful examples to use machine-learning-assisted AH-MR phase diagrams for searching the parameter spaces conducive to LMR and QAHE offer critical guidance for designing new LMR and QAHE systems.

**Figure 6.**
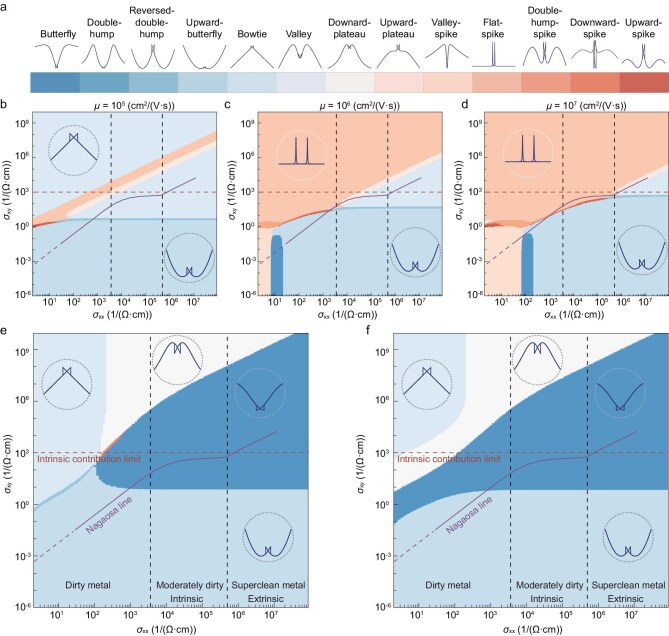
AH-MR phase diagrams with varying ${\sigma }_{\rm xx}$ and ${\sigma }_{\rm xy}$. Note that when *B* equals to 0, ${\sigma }_{\rm xy} = \sigma _{\rm xy}^{\mathrm{A}}$ and ${\sigma }_{\rm xx} = {\sigma }_1 + {\sigma }_2$. (a) Color bar showing the color representation of different AH-MR types. (b) AH-MR phase diagram where both carriers are holes. The carrier mobilities are ${\mu }_1 = {10}^5\ $(cm^2^/(V·s)) and ${\mu }_2 = {10}^3\ $(cm^2^/(V·s)), and the carrier with varying conductivity has the larger mobility. The purple line represents the theoretical ${\sigma }_{\rm xy} - {\sigma }_{\rm xx}$ relation proposed by Nagaosa *et al.* [40]. The dark orange dashed line represents the limit of the intrinsic contribution. The two vertical dashed lines separate regions where different mechanisms dominate. (c) AH-MR phase diagram where both carriers are holes. The carrier mobilities are ${\mu }_1 = {10}^6\ ( {{\mathrm{c}}{{\mathrm{m}}}^2/( {{\mathrm{V}}\cdot{\mathrm{s}}} )} )\ $and ${\mu }_2 = {10}^2\ $(cm^2^/(V·s)), and the carrier with varying conductivity has the larger mobility. (d) AH-MR phase diagram where both carriers are holes. The carrier mobilities are ${\mu }_1 = {10}^7\ $(cm^2^/(V·s)) and ${\mu }_2 = {10}^2\ $(cm^2^/(V·s)), and the carrier with varying conductivity has the larger mobility. (e) AH-MR phase diagram where the carrier with varying conductivity is electron, and the carrier with fixed conductivity is hole. The carrier mobilities are ${\mu }_e = {10}^2\ $(cm^2^/(V·s)) and ${\mu }_h = {10}^4\ $(cm^2^/(V·s)). (f) AH-MR phase diagram where both carriers are electrons. The carrier mobilities are ${\mu }_{e1} = {10}^2\ $(cm^2^/(V·s)) and ${\mu }_{e2} = {10}^4\ $(cm^2^/(V·s)).

To build up connections of our AH-MR phase diagrams with the ${\sigma }_{xy} - {\sigma }_{xx}$ scaling relationship established by Nagaosa *et al*. (here denoted as Nagaosa line) [40], we present AH-MR phase diagrams as a function of ${\sigma }_{xx}$ and ${\sigma }_{xy}$ in Figs 6 and S25. One can see that in the regime with significantly small ${\sigma }_{xy}$, AH-MR primarily exhibits the *upward-butterfly* type, displaying conventional parabolic MR behavior at lower magnetic fields, corresponding to a negligible anomalous Hall effect. Two important physics implications need to be addressed if we superimpose the Nagaosa line into the AH-MR phase diagrams. On the one hand, there is always an MR-transition boundary parallel to the Nagaosa line in the extrinsic AHE regime, in which the distance between them reflects the difference in their slopes (denoting the difference as *Δ*). One can see in Figs 6a–d and S26 that *Δ* always hosts a positive value and approaches to zero when increasing carrier mobility up to ${\mu }_1 = 1 \times {10}^7$cm^2^/(V·s), indicating that the Nagaosa line could be the limit of this MR-transition boundary at a high-mobility limit. Such a high-mobility MR scenario is exactly consistent with the MR features in Weyl semimetals with massless Weyl fermions [41,42]. On the other hand, in the regions with a large anomalous Hall angle, ${\mathrm{tan}}{\theta }^{\mathrm{A}}( {B = 0} ) = \frac{{\sigma _{xy}^{\mathrm{A}}}}{{{\sigma }_{xx}}}$, the MR state predominantly exhibits four types—*bowtie, downward-plateau, butterfly* and *upward-spike*, evolving from low mobility to high mobility. Taking Co_3_Sn_2_S_2_ and (Cr, V)*_y_*(Bi_1−_*_x_*Sb*_x_*)_2−_*_y_*Te_3_ as examples, Co_3_Sn_2_S_2_ exhibits anomalous Hall angles ${\mathrm{tan}}{\theta }^{\mathrm{A}} = 0.2\sim 0.4$ [43–45] while (Cr, V)*_y_*(Bi_1−_*_x_*Sb*_x_*)_2-_*_y_*Te_3_ hosts ${\mathrm{tan}}{\theta }^{\mathrm{A}} = 1\sim 5\ $[2,37] (in principle, as the system approaches the QAHE state, ${\sigma }_{xx}$ vanishes and the anomalous Hall angle diverges [25,40]). Simultaneously the experimental AH-MR in Co_3_Sn_2_S_2_ exhibits *butterfly* type and the AH-MR in (Cr, V)*_y_*(Bi_1−_*_x_*Sb*_x_*)_2−_*_y_*Te_3_ exhibits the *upward-spike* type, consistent with the phase diagram prediction. These observations provide a new methodology to distinguish the AHE mechanisms and offer guidance in searching for new materials systems with large anomalous Hall angles.

## DISCUSSION

Although in this work we focused primarily on the AH-MR of ferromagnetic materials, the proposed framework should also be extendable to other emerging magnetic systems, such as collinear and noncollinear antiferromagnetic materials [46–48]. Given that the spin structures of antiferromagnets differ fundamentally from those of saturated ferromagnets, the parameter ${\mathrm{\sigma }}_{xy}^{\mathrm{A}}$ requires modification to properly reflect the MR behavior in such materials. In noncollinear antiferromagnets, complex spin textures intrinsically break parity-time symmetry, generate a nonvanishing Berry curvature in the presence of spin–orbit coupling, and eventually give rise to the anomalous Hall effect. While for collinear antiferromagnets, parity-time symmetry breaking generally requires collinear spin structures combined with low crystal symmetry, and this likewise produces a finite Berry curvature when spin–orbit coupling is included [49]. These effects indicate that the strength of the anomalous Hall effect changes with external magnetic field, and therefore the parameter $\sigma _{xy}^{\mathrm{A}}$ should incorporate field dependence, such as the linear field dependence of $\sigma _{xy}^{\mathrm{A}}$ in noncollinear antiferromagnet Mn_3_Sn [50]. By including appropriate field-dependent terms in $\sigma _{xy}^{\mathrm{A}}$—such as $\sigma _{xy}^{\mathrm{A}}{\mathrm{\ }}( B ) = \sigma _{xy}^{\mathrm{A}}{\mathrm{\ }}( 0 ) + \alpha B$, where $\alpha $ is a linear coefficient—our framework can be straightforwardly extended to analyze such systems in a more precise manner.

Since our analysis is based on the two-band model, a semiclassical model that describes electronic transport by considering the two most dominant energy bands, which accounts only for the contributions of carriers in the two bands [25,26] and does not incorporate the effects of topological edge states and the magnetic-domain distributions, it cannot capture the MR behavior associated with domain reversal near the coercive fields, nor can it describe the contribution of domain edge states. This is particularly important for QAHE, where the two-band model can only reproduce the characteristic double-peak AH-MR rather than achieving a quantitative fitting for the QAHE. Specifically, in the QAHE, both the distribution of magnetic domains and their edge states play a crucial role in electrical conductivity

[9,37]. Each domain during domain reversal hosts dissipationless edge states, and electron scattering between different domains leads to finite resistance. Consequently, the two-band model cannot adequately capture the experimental QAHE behavior.

## CONCLUSION

In summary, we developed a machine-learning-assisted approach for architecting AH-MR phase diagrams within a high-dimensional parameter space. The 13 types of AH-MR states we identified cover all feasible AH-MR curves in practical ferromagnetic materials, simplifying AH-MR research for the community. The obtained phase diagrams and topological relationship networks clarify the roles of electronic parameters in AH-MR transitions and provide designable strategies to effectively manipulate AH-MR related phenomena. Our work demonstrates the potential of machine learning as a transformative tool for probing frontiers in magnetoresistance research—from unraveling magnetic anisotropy [29,51], decoding extreme magnetoresistance [52–54] to understanding (fractional) QAHE [2,3,8–11,37]. Building on this versatility, the proposed methodology further inspires the discoveries in strongly correlated systems such as topological Mott insulators [55], superconducting junctions [56,57] and Moiré superlattice structures [58] with machine-learning-assist methods.

## METHODS

### Two-band model for electron motion

The two-band model describing the electronic transport phenomena^6^ in two-dimensional systems establishes a relationship between the current density components along two orthogonal directions and the corresponding electric field components via an electrical conductivity tensor. Within this framework, the anomalous Hall conductivity $\sigma _{xy}^{\mathrm{A}}$ serves as a key parameter quantifying the strength of the anomalous Hall effect in a given material. To incorporate this effect, the $\sigma _{xy}^{\mathrm{A}}$-integrated two-band model is formulated by introducing $\sigma _{xy}^{\mathrm{A}}$ into the off-diagonal elements of the conductivity tensor, yielding the following expression (more details in Note S7):


(1) \begin{eqnarray*}
\left( {\begin{array}{@{}*{1}{c}@{}} {{J}_x}\\
{{J}_y} \end{array}} \right) = \left( {\begin{array}{@{}*{2}{c}@{}} {\displaystyle\frac{{{\sigma }_1}}{{1 + {{\left( {{\mu }_1B} \right)}}^2}} + \displaystyle\frac{{{\sigma }_2}}{{1 + {{\left( {{\mu }_2B} \right)}}^2}}}&\quad {\displaystyle\frac{{{\sigma }_1{\mu }_1B}}{{1 + {{\left( {{\mu }_1B} \right)}}^2}} + \displaystyle\frac{{{\sigma }_2{\mu }_2B}}{{1 + {{\left( {{\mu }_2B} \right)}}^2}} + \sigma _{xy}^{\mathrm{A}}}\\
{ - \displaystyle\frac{{{\sigma }_1{\mu }_1B}}{{1 + {{\left( {{\mu }_1B} \right)}}^2}} - \displaystyle\frac{{{\sigma }_2{\mu }_2B}}{{1 + {{\left( {{\mu }_2B} \right)}}^2}} - \sigma _{xy}^{\mathrm{A}}}&\quad {\displaystyle\frac{{{\sigma }_1}}{{1 + {{\left( {{\mu }_1B} \right)}}^2}} + \displaystyle\frac{{{\sigma }_2}}{{1 + {{\left( {{\mu }_2B} \right)}}^2}}} \end{array}} \right)\left( {\begin{array}{@{}*{1}{c}@{}} {{E}_x}\\
{{E}_y} \end{array}} \right),
\end{eqnarray*}

where ${J}_x$ (${J}_y$) represents the current density component along (perpendicular to) the source-drain direction in Fig. 1a, while ${E}_x$ and ${E}_y$ are the corresponding electric field components. The electrical conductivities of two charge carriers are denoted by ${\sigma }_1$ and ${\sigma }_2$, and their mobilities are given by ${\mu }_1$ and ${\mu }_2$. By inverting the electrical conductivity tensor, we would get the expression for the magnetoresistance ($\mu = \frac{\sigma }{{ne}}$):


(2) \begin{eqnarray*}
{\rho }_{xx} = \frac{{{\sigma }_1 + {B}^2{\mu }_2^2{\sigma }_1 + {\sigma }_2 + {B}^2{\mu }_1^2{\sigma }_2}}{{\left( {1 + {B}^2{\mu }_2^2} \right){\sigma }_1^2 + 2{\sigma }_1\left( {{\sigma }_2 + {B}^2{\mu }_1{\mu }_2{\sigma }_2 + B{\mu }_1\left( {1 + {B}^2{\mu }_2^2} \right)\sigma _{xy}^{\mathrm{A}}} \right) + \left( {1 + {B}^2{\mu }_1^2} \right)\left( {{\sigma }_2^2 + 2B{\mu }_2{\sigma }_2\sigma _{xy}^{\mathrm{A}} + \left( {1 + {B}^2{\mu }_2^2} \right)\sigma {{_{xy}^{\mathrm{A}}}}^2} \right)}}.\!\!\!\!\!\!\!\!\!\! \\
\end{eqnarray*}

Technically, this expression represents the resistivity, which is related to the experimentally measured resistance ${R}_{xx}$ via the formula ${R}_{xx} = {\rho }_{xx}\frac{L}{w}$. Here, *L* denotes the length of the Hall bar device along the direction of the applied current, while *w* corresponds to the width perpendicular to the current flow. This expression contains five parameters, ${\sigma }_1$, ${\sigma }_2$, $\sigma _{xy}^{\mathrm{A}}$, ${\mu }_1$ and ${\mu }_2$. This multi-parameter system benefits from the data scaling capability of machine learning.

To account for the observed hysteresis in the conductivity ${\sigma }_{xx}$ (the first-row, first-column component of the electrical conductivity tensor) with respect to the external magnetic field, a correction term $\alpha $ was phenomenologically introduced into the two-band model:


(3) \begin{eqnarray*}
\left( {\begin{array}{@{}*{1}{c}@{}} {{J}_x}\\
{{J}_y} \end{array}} \right) = \left( {\begin{array}{@{}*{2}{c}@{}} {\displaystyle\frac{{{\sigma }_1}}{{1 + {{\left( {{\mu }_1\left( {B + \alpha \sigma _{xy}^{\mathrm{A}}} \right)} \right)}}^2}} + \displaystyle\frac{{{\sigma }_2}}{{1 + {{\left( {{\mu }_2\left( {B + \alpha \sigma _{xy}^{\mathrm{A}}} \right)} \right)}}^2}}}&\quad\!\!\! {\displaystyle\frac{{{\sigma }_1{\mu }_1B}}{{1 + {{\left( {{\mu }_1B} \right)}}^2}} + \displaystyle\frac{{{\sigma }_2{\mu }_2B}}{{1 + {{\left( {{\mu }_2B} \right)}}^2}} + \sigma _{xy}^{\mathrm{A}}}\\
{ - \displaystyle\frac{{{\sigma }_1{\mu }_1B}}{{1 + {{\left( {{\mu }_1B} \right)}}^2}} - \frac{{{\sigma }_2{\mu }_2B}}{{1 + {{\left( {{\mu }_2B} \right)}}^2}} - \sigma _{xy}^{\mathrm{A}}}&\quad\!\!\! {\displaystyle\frac{{{\sigma }_1}}{{1 + {{\left( {{\mu }_1\left( {B + \alpha \sigma _{xy}^{\mathrm{A}}} \right)} \right)}}^2}} + \displaystyle\frac{{{\sigma }_2}}{{1 + {{\left( {{\mu }_2\left( {B + \alpha \sigma _{xy}^{\mathrm{A}}} \right)} \right)}}^2}}} \end{array}} \right)\left( {\begin{array}{@{}*{1}{c}@{}} {{E}_x}\\
{{E}_y} \end{array}} \right).\!\!\!\!\\
\end{eqnarray*}

Note that the sign of $\sigma _{xy}^{\mathrm{A}}$ reverses whenever the magnetization direction flips, regardless of whether the change is from up to down or down to up. This property makes the additional term $\alpha \sigma _{xy}^{\mathrm{A}}$ particularly well-suited for characterizing the hysteresis behavior. Similarly, by inverting the tensor in Equation (3), the expression for ${\rho }_{xx}$ can be derived. The fitting results presented in this article are based on this modified two-band model. In this study, every MR curve comprises two segments: the left segment spans from −1 T to 0.06 T and corresponds to $\sigma _{xy}^{\mathrm{A}}$, while the right segment spans from 1 T to −0.06 T and corresponds to −$\sigma _{xy}^{\mathrm{A}}$. This means the coercive field is set to be 0.06 T.

### Machine learning methods

A total of 2274 700 parameter sets (${n}_1$, ${n}_2$, ${\sigma }_1$, ${\sigma }_2$, $\sigma _{xy}^{\mathrm{A}}$) were randomly sampled within the following ranges: from 10^12^ cm^−2^ to 10^15^ cm^−2^ for$\ | {{n}_1} |$ and$\ | {{n}_2} |;$ and from 10^−5^ S to 1 S for ${\sigma }_1$, ${\sigma }_2$ and $\sigma _{xy}^{\mathrm{A}}$. For each set, the corresponding magnetoresistance state were generated. Since the analysis focuses on the shape of the MR curves rather than their absolute resistivity values, each ${\rho }_{xx}( B )$ curve was normalized to the range between 0 and 1. These MR curves were then represented as points in a 531-dimensional space and clustered using the mean-shift algorithm (see Note S2 for details). We employed the scaled exponential linear unit activation function, which promotes stable training by normalizing layer outputs toward zero mean and unit variance [59]. For training, approximately 110 MR instances per curve type were selected, reserving 20% as a validation set and using the remainder for training. The model was implemented in Wolfram Mathematica, and the code is provided in Note S3.

The heatmap and the topological networks were constructed based on the following procedure. Since each AH-MR curve is generated by inputting values for the five parameters into the two-band model, each of these feasible AH-MR curves can be considered as a point in the 5-dimensional parameter space. We first randomly sampled ∼45 000 points in this space, then defined 242 neighboring points for each by varying the five parameters. This process yielded over 10 million data points, which were used to categorize their corresponding AH-MR states. Whenever the AH-MR type of a sampled point differed from that of a neighboring point, we recorded the parameter differences and state transition as a single event. For example, a typical transition from a *bowtie* to a *butterfly* AH-MR involved the following parameter changes: ${n}_1\ $changes from 6.84 × 10^12^ cm^−2^ to 6.93 × 10^12^ cm^−2^; ${n}_2$ changes from 1.49 × 10^12^ cm^−2^ to 1.40 × 10^12^ cm^−2^; ${\sigma }_1$ remains unchanged; ${\sigma }_2\ $changes from 0.031 S to 0.025 S; $\sigma _{xy}^{\mathrm{A}}$ changes from 0.001 S to 0.006 S. After processing all 45 000 sampled points and their neighbors, we recorded a total of 688 050 transitions. This comprehensive dataset allowed us to construct a detailed network topology, accurately representing transition probabilities (denoted as correlation coefficients) between each pair of AH-MR states.

### Device fabrication and electronic transport measurement

The Fe_5_GeTe_2_ flakes were first exfoliated onto PDMS (polydimethylsiloxane) and then transferred onto SiO_2_/Si substrates with Ti/Au (3/12 nm) Hall-bar electrodes prepatterned by a standard electron beam lithography process. Then, the devices were immediately spin-coated with PMMA (polymethyl methacrylate) for protection from air. All the performed exfoliation and transfer processes were performed inside a N_2_ gas glovebox (oxygen and water levels below 0.1 ppm). A solution composed of LiClO_4_ and PEO is used as the gating electrolyte, which is created by dissolving a mixture of LiClO_4_ and PEO powders in the anhydrous methanol solvent. Longitudinal and Hall voltages were measured with synchronized lock-in amplifiers (SR830, Stanford Research Systems) in an Oxford Instruments Teslatron^PT^ system. Gate voltages were applied with a sweep rate of 0.5 mV s^–1^ using a direct current source meter (Keithley 2400). The devices were directly zero-field cooled from 300 K to 1.5 K. We then increased the temperature, and electronic measurements were conducted during this warm-up process. Refer to Note S9 for more information on device fabrication and electronic transport measurement.

## Supplementary Material

nwag228_Supplemental_File

## Data Availability

The data that support the plots within this paper and other findings of this study are available from the corresponding author upon reasonable request.
