# Supplementary material for: Machine-learning-based architecting of magnetoresistance phase diagrams in anomalous Hall systems
Source: Natl Sci Rev. 2026 Apr 20;13(13):nwag228. doi: 10.1093/nsr/nwag228 (PMC13458612; doi:10.1093/nsr/nwag228)
Supplement: Supplementary file 1 [file nwag228_supplemental_file.docx]

**Supplementary Information for**

**Machine-Learning-Based Architecting of Magnetoresistance Phase Diagrams in Anomalous Hall Systems**

Ganyu Chen ^1†^, Xiangyu Bi^1†^, Zeya Li^2†^, Xiao Feng^3^, Yang Feng^4^, Feng Qin^1^, Junwei Huang^1^, Ling Zhou^2^, Ke He^3^, Toshiya Ideue^5^, Qi-Kun Xue^2,3,6^*, Hongtao Yuan^1,7^*

^1^*National Laboratory of Solid State Microstructures, College of Engineering and Applied Sciences, Collaborative Innovation Center of Advanced Microstructures, Nanjing University, Nanjing 210093, China.*

^2^*Quantum Science Center of Guangdong-Hong Kong-Macao Greater Bay Area, Shenzhen 518045, China*

^3^*State Key Laboratory of Low Dimensional Quantum Physics and Department of Physics, Tsinghua University, Beijing 100084, China.*

^4^*Beijing Academy of Quantum Information Sciences, Beijing 100193, China*

^5^*Quantum Phase Electronic Center and Department of Applied Physics, The University of Tokyo, Tokyo 113-8654, Japan.*

^6^*Department of Physics and Guangdong Basic Research Center of Excellence for Quantum Science, Southern University of Science and Technology, Shenzhen 518055, China*

^7^*Jiangsu Physical Science Research Center, Nanjing 210093, China*

E-mail: [htyuan@nju.edu.cn](mailto:htyuan@nju.edu.cn) (H.T.Y.), [qkxue@mail.tsinghua.edu.cn](mailto:%20qkxue@mail.tsinghua.edu.cn) (Q.K.X.).

^†^ These authors contributed equally to this work.

**Outline:**

1. **Workflow of our machine learning analysis approach**
2. **Density-based clustering for MR curves with the mean-shift algorithm**
3. **Self-normalizing neural network (SNN) for MR curve recognition**
4. **AH-MR phase diagrams as a function of conductivity and carrier density**
5. **Generated MR curves and phase diagrams**
6. **Relationships between AH-MR states and electronic parameters**
7. **AH-MR curve fitting with the two-band model**
8. **Comparison between the revised model and the empirical relation**
9. **Transport measurement for gated Fe_5_GeTe_2_ devices**
10. **Two-band model analysis for MR data of gated devices**
11. **Machine learning prediction of GMR**
12. **Machine learning prediction of QAHE-like magnetoresistance**
13. **AH-MR phase diagrams with varying** $\boldsymbol{\sigma}_{\boldsymbol{xx}}$ **and** $\boldsymbol{\sigma}_{\boldsymbol{xy}}$
14. **Possible physical origin of different AH-MR states**
1. **Workflow of our machine learning analysis approach**

The workflow of our approach is illustrated in Supplementary Fig. 1. We first used the 5-parameter expression two-band model for AH-MR to generate about 2,274,700 MR curves by varying electrical conductivities and carrier densities of the two carriers as well as the anomalous Hall conductivity. The expression for $\rho_{xx}$ is

$$\rho_{xx}=\frac{\sigma_{1}+B^{2}{\mu_{2}}^{2}\sigma_{1}+\sigma_{2}+B^{2}{\mu_{1}}^{2}\sigma_{2}}{\left( 1+B^{2}{\mu_{2}}^{2} \right){\sigma_{1}}^{2}+2\sigma_{1}\left( \sigma_{2}+B^{2}\mu_{1}\mu_{2}\sigma_{2}+B\mu_{1}\left( 1+B^{2}{\mu_{2}}^{2} \right)\sigma_{xy}^{A} \right)+\left( 1+B^{2}{\mu_{1}}^{2} \right)\left( {\sigma_{2}}^{2}+2B\mu_{2}\sigma_{2}\sigma_{xy}^{A}+\left( 1+B^{2}{\mu_{2}}^{2} \right){\sigma_{xy}^{A}}^{2} \right)}$$

(Equation (2) in the main text). Next, we mapped each curve to a point in a 531-dimensional space and applied the density-based clustering algorithm mean shift to group these points into distinct categories. From each cluster, we selected representative data to construct a training dataset, which was then used to train a neural network for classifying AH-MR states based on the identified clusters. To ensure self-normalization, we employed the scaled exponential linear unit (SELU) as the activation function. Finally, to explore the relationships between AH-MR states and electronic parameters, we analyzed a large ensemble of MR curves generated under varying parameter combinations. The trained neural network predicted the corresponding AH-MR state types, enabling systematic studies. While machine learning serves as a powerful tool for classification in this work, its essential role is to enable a comprehensive exploration of a high-dimensional parameter space. This approach allows for the discovery of distinct, universal classes of AH-MR behavior that form the foundation for subsequent physical interpretation. The ML framework is a guide for uncovering underlying mechanisms, transforming a computational tool into a driver of physical insight. Details for each step are provided in Supplementary Notes 2-7. For the generation of the dataset, the steps are given as follows:

1) **Define the parameter space.** We first specified the ranges for the five parameters as mentioned above.
2) **Randomly generate parameter sets.** We used the function “RandomReal” in Mathematica to generate values for the five parameters within their corresponding ranges.
3) **Generate MR curves.** Randomly generated parameter sets were then used as inputs to the two-band model, from which the corresponding MR curves were obtained.
4) **Construct the dataset.** We aggregated all generated curves to form the final dataset.

Supplementary Figure 1. Workflow of our machine learning approach. First, we use the 5-parameter expression for AH-MR to generate a large set of MR curves. Next, we apply density-based clustering to group these curves into distinct categories. From each category, we select representative data to form a training dataset, which is then used to train a neural network capable of automatically classifying MR curves based on the clusters identified in the previous step. Finally, utilizing the trained neural network, we explore the relationships between AH-MR states and electronic parameters.

2. **Density-based clustering for MR curves with the mean-shift algorithm**

In general, machine learning refers to the process where the computer learns from training data and subsequently generates a model that can make predictions based on unseen data [1,2]. Machine learning can be categorized into two main types: supervised learning and unsupervised learning. In supervised learning, the algorithm learns the relationship between independent variables (features) and a dependent variable (target) using labeled training data—where the correct outputs are provided. Once trained, the model can predict the dependent variable for new, unseen inputs. For instance, given training data $\left( \boldsymbol{x}_{\boldsymbol{1}},y_{1} \right)$,$\left( \boldsymbol{x}_{\boldsymbol{2}},y_{2} \right)$,…, $\left( \boldsymbol{x}_{\boldsymbol{n}},y_{n} \right)$, where each $\boldsymbol{x}_{i}$ may be a feature vector (i.e., multiple input variables) and $y_{i}$ is the corresponding label, the goal is to derive a function $f(\boldsymbol{x}_{\boldsymbol{i}})$ such that $f\left( \boldsymbol{x}_{\boldsymbol{i}} \right)\approx y_{i}$. This model can then generalize to predict $y$ for new $x$ values not present in the training set. In contrast, unsupervised learning deals with unlabeled data $\left( \boldsymbol{x}_{\boldsymbol{1}} \right)$,$\left( \boldsymbol{x}_{\boldsymbol{2}} \right)$,…, $\left( \boldsymbol{x}_{\boldsymbol{n}} \right)$, where no predefined outputs are provided. Here, the algorithm autonomously identifies underlying structures, such as clusters or patterns, grouping similar data points together based on their features.

In this work. we employed the unsupervised machine learning method mean-shift to cluster MR curves. Mean shift is a density-based clustering method and aims to find clusters by iteratively shifting data points towards regions of higher density [3]. The formula for iteration is

$$\begin{aligned} \boldsymbol{x}^{\boldsymbol{'}}=\frac{\sum_{i} ⅇ^{-||\boldsymbol{x}_{\boldsymbol{i}}-\boldsymbol{x}{||}^{2}/\epsilon^{2}}}{\sum_{i} ⅇ^{-||\boldsymbol{x}_{\boldsymbol{i}}-\boldsymbol{x}{||}^{2}/\epsilon^{2}}}\boldsymbol{x}_{\boldsymbol{i}}\#\left( 1 \right) \end{aligned}$$

where $\boldsymbol{x}_{\boldsymbol{i}}$ is the coordinate of the *i*^th^ data point; $\boldsymbol{x}$ is the coordinate of the data point under investigation and $\epsilon$ is called the effective neighborhood radius. The mean shift algorithm performs Equation ($1$) on all the data points iteratively until the position of all the points stop changing. For example, Supplementary Figure 2a shows a set of two-dimensional data points. If we perform mean shift clustering on them, the data points would move closer and closer towards the regions with higher data density of points. The results of different iterations are shown in Supplementary Fig. 2b. Finally, all the data points that stop moving at the same position are classified into the same cluster. As can be seen from Supplementary Fig. 2b, there were in the end 4 positions where data points were gathered, meaning there were four clusters and the original data points which moved to the same one of the four positions belonged to the same cluster, as illustrated in Supplementary Fig. 2c.

Normally, the electrical conductivities of ferromagnetic metals, such as MnSi, Fe_5_GeTe_2_, Cu_1-_*_x_*Zn*_x_*Cr_2_Se_4_ and La_1-_*_x_*Sr*_x_*CoO_3_ [4,5], are about $1\times{10}^{6}\mathrm{to}1\times{10}^{7}$S/m, which means that for typical metallic two-dimensional samples with a length of several micrometers, a width of several micrometers and a thickness of several tens of nanometers, the sheet conductivity is approximately on the order of 0.01 S to 1 S. While normally for magnetic semiconductors, such as Ga_1-_*_x_*Mn*_x_*As, Cd_1-_*_x_*Mn*_x_*Te and Hg_1-_*_x_*Mn*_x_*Te, the electrical conductivities are approximately on the order of $1\times{10}^{3}$S/m [6-8], which means that for typical semiconducting two-dimensional samples, the electrical conductivity is approximately on the order of $1\times{10}^{-5}$ S to $1\times{10}^{-4}$ S. Therefore, the reasonable conductivity range in our two-band model for AH-MR analysis is set from $1\times{10}^{-5}$ S to 1 S.

It is sensible to hypothesize that AH-MR curves exhibiting similar shapes belong to the same category. To formalize this idea, we first generated 2,274,700 MR curves, and then discretized each MR curve by evaluating $\rho_{xx}(B)$ at 531 predefined $B$ values, resulting in a vector representation of each curve comprising 531 $\rho_{xx}$ values. This discretization effectively maps each curve as a point in a 531-dimensional space. Specifically, the 2,274,700 MR curves were clustered using the mean-shift method on Wolfram Mathematica, consequently obtaining 22 clusters of AH-MR states. After careful inspection of these obtained clusters, we identified 13 distinct AH-MR state types. The obtained AH-MR types and their percentage in the 2,274,700 MR curves are given in Supplementary Fig. 3 and Supplementary Table 1.

**Supplementary Table 1.** AH-MR types and their percentages in the data used for clustering

| AH-MR type | Number of curves | Percentage (%) |
| --- | --- | --- |
| Bowtie | 661,626 | 29.09 |
| Flat-spike | 461,977 | 20.31 |
| Butterfly | 447,071 | 19.65 |
| Upward-butterfly | 294,686 | 12.95 |
| Valley | 92,814 | 4.08 |
| Upward-plateau | 92,361 | 4.06 |
| Double-hump | 90,234 | 3.97 |
| Valley-spike | 51,892 | 2.28 |
| Downward-spike | 32,193 | 1.42 |
| Reversed-double-hump | 24,668 | 1.08 |
| Double-hump-spike | 16,187 | 0.71 |
| Downward-plateau | 7,451 | 0.33 |
| Upward-spike | 1,540 | 0.07 |

Supplementary Figure 2. An example of the density-based clustering method mean shift. (a) Initial data points in a two-dimensional space. (b) Data points after certain iterations by mean shift. (c) Clustering results. Data points classified into the same category share the same color.

Supplementary Figure 3. Pie chart illustrating the obtained AH-MR states and their percentages. The names and percentages of these 13 AH-MR types are given below their corresponding shapes.

3. **Self-normalizing neural network (SNN) for MR curve recognition**

**A brief introduction to neural network.** A neural network is a supervised learning algorithm inspired by the biological nervous system. In biological systems, neurons are interconnected, and when a neuron is activated, it transmits chemical signals to neighboring neurons. If the total input received by a neuron exceeds a certain threshold, it becomes activated and propagates signals to other neurons. Similarly, the fundamental unit of an artificial neural network—also called a neuron—receives input data, processes it through a mathematical operation, and passes the output to subsequent neurons. A simple neural network architecture, consisting of only one hidden layer (any layer between the input and output layers) with a single neuron, is illustrated in Supplementary Fig. 4a. The neural network is trained on a dataset comprising independent variables $x_{1}$, $x_{2}$, along with a dependent variable $y$. Once trained, the network predicts the value of $y$ based on the input data. The hidden layer consists of a single neuron, $\alpha_{1}$. When input data $x_{1}$ and $x_{2}$ are provided, the neuron computes the function $f(\sum_{i=1}^{n} w_{i}x_{i}-\theta)$ where $w_{1}$ and $w_{2}$ are the weights, $\theta$ is the threshold, and the output of $f$ serves as the predicted value of $y$. For instance, if $w_{1}=0.8,w_{2}=0.2,\theta=0.5,f(\sum_{i=1}^{n} w_{i}x_{i}-\theta)=\{\begin{matrix} 1 & \sum_{i=1}^{n} w_{i}x_{i}-\theta>0 \\ 0 & \sum_{i=1}^{n} w_{i}x_{i}-\theta<0 \end{matrix}$, and given input values $x_{1}=0.3$ and $x_{2}=0.1$, the computation yields $f\left( \sum_{i=1}^{n} w_{i}x_{i}-\theta\right)=f\left( 0.8\times0.3+0.2\times0.1-0.5 \right)=f\left( -0.24 \right)=0$. Thus, the model predicts $=0$ for these inputs.

Typically, neural networks consist of many layers of neurons, with each layer of neurons connected to the previous layer by a certain function $f$, which is called an activation function, as shown in Supplementary Fig. 4b. In the figure, $x_{1}$ and $x_{2}$ represent two independent variables. $w_{(i)(j)(k)}$ represents the weight in the activation function between the *j*^th^ neuron in the *i*^th^ layer and the *k*^th^ neuron in the (i+1) ^th^ layer. $b_{(i)(k)}$ represents the threshold of the *k*^th^ neuron in the (*i*+1) ^th^ layer. The process begins by computing the activation function values from the input variables $x_{1}$ and $x_{2}$. These values are then propagated forward to the neurons in the subsequent layer. This forward propagation continues iteratively, with each layer processing the outputs of the preceding layer, until the final layer generates the predicted output $y$.

During training, the model learns from a provided dataset by iteratively adjusting all weights and biases to minimize prediction error. This optimization is achieved through backpropagation, an algorithm that efficiently computes gradients for parameter updates. The underlying principle of backpropagation can be elucidated through the following example.

Let $\alpha_{(i)(j)}$ represent the output value of the *j*^th^ neuron in the *i*^th^ layer. Consider a neural network with n layers, where the final (output) layer consists of 3 neurons. Initially, all weights and thresholds in the model are randomly initialized. After a set of training data is provided and processed through the neural network, the output values of the three neurons in the last layer are $\alpha_{\left( n \right)\left( 1 \right)}=0.3$，$\alpha_{\left( n \right)\left( 2 \right)}=0.25$, and $\alpha_{\left( n \right)\left( 3 \right)}=0.2$. However, the real values for these neurons in the training data are $\alpha_{(n)(1)}^{\mathrm{real}}=0.35$，$\alpha_{(n)(2)}^{\mathrm{real}}=0.1$, and $\alpha_{(n)(3)}^{\mathrm{real}}=0.15$. To minimize the prediction error, the model must adjust its outputs such that: $\alpha_{\left( n \right)\left( 1 \right)}$ needs to be increased, $\alpha_{\left( n \right)\left( 2 \right)}$ needs to be decreased, and $\alpha_{\left( n \right)\left( 3 \right)}$ needs to be increased. This adjustment requires modifying the weights and thresholds in the preceding layers to propagate the necessary changes. For instance, suppose the ($n$−1)^th^ layer contains 2 neurons, the parameters influencing $\alpha_{\left( n \right)\left( 1 \right)}$ include $w_{(n-1)(1)(1)}$, $b_{(n-1)(1)}$, and$w_{(n-1)(2)(1)}$. If we define $\boldsymbol{\beta}_{(n)(1)}=\left( w_{\left( n-1 \right)\left( 1 \right)\left( 1 \right)},b_{\left( n-1 \right)\left( 1 \right)},w_{\left( n-1 \right)\left( 2 \right)\left( 1 \right)},\alpha_{\left( n-1 \right)\left( 1 \right)},\alpha_{\left( n-1 \right)\left( 2 \right)} \right)$, then $\alpha_{\left( n \right)\left( 1 \right)}$ is a function of the parameter $\boldsymbol{\beta}_{(n)(1)}$. Calculating its gradient $\nabla_{\boldsymbol{\beta}(n)(1)}\alpha_{\left( n \right)\left( 1 \right)}$ will reveal the direction of fastest change for $\alpha_{\left( n-1 \right)\left( 1 \right)}$. Similarly, $\alpha_{\left( n-1 \right)\left( 1 \right)}$ is also a function of the weights and thresholds of the previous layer's neurons. Therefore, in $\nabla_{\boldsymbol{\beta}(n)(1)}\alpha_{\left( n \right)\left( 1 \right)}$, $\alpha_{\left( n-1 \right)\left( 1 \right)}$ and $\alpha_{\left( n-1 \right)\left( 2 \right)}$ can be replaced with the weights and thresholds from the previous layer's neurons. This process is repeated recursively until all neuron output values in $\nabla_{\boldsymbol{\beta}(n)(1)}\alpha_{\left( n \right)\left( 1 \right)}$ are converted into weights and thresholds. Once the gradient is represented solely in terms of these parameters, we can adjust them by taking a step in the direction of the gradient. This iterative optimization process gradually aligns the output of the final layer with the desired target values.

The previous discussion focused on adjusting the neuron $\alpha_{\left( n \right)\left( 1 \right)}$ in the final layer. In practice, however, this adjustment should be applied to all three neurons—$\alpha_{\left( n \right)\left( 1 \right)}$, $\alpha_{\left( n \right)\left( 2 \right)}$, and $\alpha_{\left( n \right)\left( 3 \right)}$—by performing the same operations on each and then averaging their respective weight and threshold updates. The training process involves iteratively executing this backpropagation algorithm until the loss function is minimized. The loss function quantifies how well the model fits the training data, with mean squared error being a common choice. Mean squared error computes the average squared difference between the model’s predictions and the true target values from the dataset. In short, back-propagation aims to find the parameter values that minimize the loss function. Supplementary Figure 4c illustrates this process, showing how the loss function reaches its minimum by following the gradient descent method within a parameter space spanned by two parameters $R_{x}$ and $R_{y}$.

**The development of our neural network model.** To automatically categorize an AH-MR curve into one of the 13 types, we trained a self-normalizing neural network (SNN) consisting of one input layer, 8 hidden layers and one output layer. The activation function we chose is the scaled exponential linear unit function. This function introduces self-normalizing properties and is able to mitigate the issue of vanishing or exploding gradients in neural network training [9]. The mathematical expression of the scaled exponential linear unit function is

$$\left\{ \begin{aligned} 1.0507x, x\geq0 \\ 1.7581\left( ⅇ^{x}-1 \right), x<0 \end{aligned} \right.$$

For each type of AH-MR curves, we carefully selected about 110 MR curves as the training dataset, of which 20 percent was the validation set while the remainder is the training set. The confusion matrices separately for the validation dataset and the training dataset are shown in Supplementary Fig. 5.

**Supplementary Figure** **4.** Key concepts of neural network. (a) A simple neural network consisting of one hidden layer which has only one neuron. (b) Illustration of a general neural network. (c) Illustration of gradient descent in back propagation.

**Supplementary Figure** **5.** Normalized confusion matrix for the trained SNN. (a) Confusion matrix demonstrating the performance of the trained SNN when evaluated on the validation dataset. (b) Confusion matrix demonstrating the performance of the trained SNN when evaluated on the training dataset.

4. **AH-MR phase diagrams as a function of conductivity and carrier density**

To illustrate the influence of electrical conductivity on the AH-MR type, we present several phase diagrams to depict the evolution of AH-MR behavior with varying carrier conductivities (Supplementary Figs. 6-8). Supplementary Figure 6 displays phase diagrams for systems where both carriers are holes at different densities. When both hole densities are low, the AH-MR type exhibits greater diversity, whereas at high densities, the MR curves predominantly adopt a *bowtie* shape. Supplementary Figure 7 presents phase diagrams for systems with two electron carriers. Here, the MR curves show minimal variation across different electron densities, largely retaining a *butterfly* shape. Supplementary Figure 8 explores the case where one carrier is an electron and the other is a hole. Each phase diagram is approximately bisected into two distinct regions: one where the AH-MR type is *butterfly*-shaped and another where it is not. When the hole density significantly exceeds the electron density, the *butterfly* type dominates. Conversely, at much higher electron densities, *bowtie* and *upward-butterfly* types become more prevalent. However, when electron and hole densities are comparable, the phase diagram divides nearly equally between *butterfly* and *bowtie* types.

**Supplementary Figure** **6.** Phase diagrams illustrating the AH-MR type as a function of electrical conductivities when both carriers are holes. Each small panel shows the AH-MR type evolution with electrical conductivities, and a total of 10 phase diagrams obtained at different carrier concentrations are given.

**Supplementary Figure** **7.** Phase diagrams illustrating the AH-MR type as a function of electrical conductivities when both carriers are electrons. Each small panel shows the AH-MR type evolution with electrical conductivities, and a total of 10 phase diagrams obtained at different carrier concentrations are given.

**Supplementary Figure** **8.** Phase diagrams illustrating the AH-MR type as a function of electrical conductivities when one carrier is electron, and the other carrier is hole. Each small panel shows the AH-MR type evolution with electrical conductivities, and a total of 16 phase diagrams obtained at different carrier concentrations are given.

5. **Generated MR curves and phase diagrams**

To provide clear visualization of how AH-MR type evolves with carrier densities, we provide phase diagrams containing small panels showing actual MR curves. Each small panel’s background color corresponds to the color representation used in Fig. 3 in the main text. Supplementary Figure 9 corresponds to Fig. 3a in the main text, and it can be seen that curves with spikes are more likely to be found in this high conductivity regime. Supplementary Figure 10 corresponds to Fig. 3d in the main text, and it can be seen that the curve type is less diverse in this low conductivity regime. Supplementary Figures 11 and 12 correspond to the Fig. 3e and Fig. 3h in the main text, respectively. It can be seen that the carrier with higher electrical conductivity has plays a more important role in determining the behavior of MR.

In general, the anomalous Hall conductivity can be either positive or negative, depending on the Berry curvature of the occupied energy bands near the Fermi level [10-12]. To investigate how the sign of the anomalous Hall conductivity influences the AH-MR curve type, we constructed phase diagrams under identical electrical conductivity conditions as in Fig. 3a-h, but with the anomalous Hall conductivity values inverted (see Supplementary Fig. 13). Interestingly, for every phase diagram in Fig. 3, reversing the sign of the anomalous Hall conductivity results in a phase diagram that is a 180° rotation of the original phase diagram around its center. This observation indicates that reversing the sign of anomalous Hall conductivity is equivalent to changing the carrier type from electron to hole or from hole to electron. This effect can also be derived from the AH-MR expression by replacing $n_{1}$ with $-n_{1}$, $n_{2}$ with ${-n}_{2}$ and $\sigma_{xy}^{A}$ with $-\sigma_{xy}^{A}$ in Equation (2) in the main text.

**Supplementary Figure** **9.** MR curves obtained at different carrier densities when $\sigma_{1}=1 S$, $\sigma_{2}=0.95 S$ and $\sigma_{\mathrm{xy}}^{A}=0.08 S$. (a) MR curves obtained when *B* ranges from -1 T to 1 T. (b) MR curves obtained when *B* ranges from -0.3 T to 0.3 T. Different background colors represent different curve types, as indicated by the color bar on the right. This figure corresponds to Fig. 3a in the main text.

**Supplementary Figure** **10.** MR curves obtained at different carrier densities when $\sigma_{1}=1\times{10}^{-3} S$, $\sigma_{2}=0.95\times{10}^{-3} S$ and $\sigma_{xy}^{A}=0.08\times{10}^{-3} S$. (a) MR curves obtained when *B* ranges from -1 T to 1 T. (b) MR curves obtained when *B* ranges from -0.3 T to 0.3 T. Different background colors represent different curve types, as indicated by the color bar on the right. This figure corresponds to Fig. 3d in the main text.

**Supplementary Figure** **11.** MR curves obtained at different carrier densities when $\sigma_{1}=1 S$, $\sigma_{2}=0.1 S$ and $\sigma_{\mathrm{xy}}^{A}=0.08 S$. (a) MR curves obtained when *B* ranges from -1 T to 1 T. (b) MR curves obtained when *B* ranges from -0.3 T to 0.3 T. Different background colors represent different curve types, as indicated by the color bar on the right. This figure corresponds to Fig. 3e in the main text.

**Supplementary Figure** **12.** MR curves obtained at different carrier densities when $\sigma_{1}=1\times{10}^{-3} S$, $\sigma_{2}=0.1\times{10}^{-3} S$ and $\sigma_{\mathrm{xy}}^{A}=0.08\times{10}^{-3} S$. (a) MR curves obtained when *B* ranges from -1 T to 1 T. (b) MR curves obtained when *B* ranges from -0.3 T to 0.3 T. Different background colors represent different curve types, as indicated by the color bar on the right. This figure corresponds to Fig. 3h in the main text.

**Supplementary Figure** **13.** Phase diagrams illustrating the MR curve type as a function of carrier densities. (a-d) Phase diagrams where the electrical conductivity of the second carrier is 0.95 times that of the first carrier. From b to d, each panel has an electrical conductivity that is one-tenth of the previous panel. In every diagram, the anomalous Hall conductivity remains at -0.08 times the electrical conductivity of the first carrier. Different colors represent different MR curve types, as indicated by the color bar at the bottom. (e-f) Phase diagrams where the electrical conductivity of the second carrier is 0.1 times that of the first carrier. Each successive panel from f to h has an electrical conductivity that is one-tenth of the previous panel, with the anomalous Hall conductivity consistently at -0.08 times the electrical conductivity of the first carrier. (i) MR curves at different carrier densities on the lines αβ and ξω in (d) αβ corresponds to $n_{2}=-2\times{10}^{12}$cm^-2^, and ξω corresponds to $n_{1}=100\times{10}^{12}$ cm^-2^.

6. **Relationships between AH-MR states and electronic parameters**

To elucidate the relationships between different AH-MR state types and the electronic transport parameters, we employed the approach described in the Methods section of the main text. The sampled points are chosen within the ranges $n_{1}\in(6.24\times{10}^{11},1\times{10}^{15})\bigcup(-1\times{10}^{15},-6.24\times{10}^{11})(\mathrm{cm}^{-2}),n_{2}\in(6.24\times{10}^{11},1\times{10}^{15})\bigcup(-1\times{10}^{15},-6.24\times{10}^{11})(\mathrm{cm}^{-2}),\sigma_{1}\in(0.001,1)(S),\sigma_{2}\in(0.001,1)(S),\sigma_{xy}^{A}\in(0.001,3)(S)$. The neighboring points in the 5-dimensional parameter space mentioned in the main text is defined as follows. A point is considered a neighbor of a sampled point if either of the following conditions holds: $\sigma_{1}$, $\sigma_{2}$, or $\sigma_{xy}^{A}$ differs by $\pm0.0025$ S from that of the sampled point, $n_{1}$ or $n_{2}$ differs from the sampled point's value by the following amounts: $\pm6.24\times{10}^{12}$ $\mathrm{cm}^{-2}$ if the sampled point's absolute value is greater $1\times{10}^{14} \mathrm{cm}^{-2}$, $\pm6.24\times{10}^{11}$ $\mathrm{cm}^{-2}$ if the sampled point's absolute value is between $1\times{10}^{12} \mathrm{cm}^{-2}$ and $1\times{10}^{13} \mathrm{cm}^{-2}$, $\pm6.24\times{10}^{10}$ $\mathrm{cm}^{-2}$ if the sampled point's absolute value is between $6.24\times{10}^{11} \mathrm{cm}^{-2}$ and $1\times{10}^{12} \mathrm{cm}^{-2}$. Therefore, each sampled point has $3^{5}-1=242$ neighboring points.

7. **AH-MR curve fitting with the two-band model**

**Semiclassical Bloch electron equations of motion in two-dimensional systems.** Assuming the effective mass of a carrier is $m$, the collision relaxation time is $\tau$, and the charge is $q$ (-$1.602\times{10}^{-19} C$ for electrons and $1.602\times{10}^{-19} C$ for holes), under the semiclassical approximation,

$$\begin{aligned} m\left( \frac{d}{\mathrm{dt}}+\frac{1}{\tau} \right)\boldsymbol{v}=q\left( \boldsymbol{E}+\boldsymbol{v}\times\boldsymbol{B} \right)\#\left( \text{}\text{}\text{}\text{2}\text{} \right) \end{aligned}$$

where $\boldsymbol{v}$ is the carrier velocity, $\boldsymbol{E}$ is the electric field, and $\boldsymbol{B}$ is the magnetic induction strength. Under the condition that *B* is perpendicular to the two-dimensional material surface and pointed upward, then when the system has reached the steady state (i.e., $\frac{d\boldsymbol{v}}{\mathrm{dt}}=0$),

$$\begin{aligned} v_{x}=\mu E_{x}-\mu Bv_{y}\#\left( \text{}\text{}\text{}\text{3}\text{} \right) \end{aligned}$$

$$\begin{aligned} v_{y}=\mu E_{y}+\mu Bv_{x}\#\left( \text{}\text{}\text{}\text{4}\text{} \right) \end{aligned}$$

where $\mu=\frac{q\tau}{m}$ is the mobility of the carrier, and $B$ is the magnitude of the magnetic induction strength. Assuming that the density of the carrier is $n$, then since the current density $\boldsymbol{J}=nq\boldsymbol{v}$**,** we have

$$\begin{aligned} v_{x}=\frac{q\tau}{m}E_{x}+\frac{\tau qB}{m}v_{y}\#\left( \text{}\text{}\text{}\text{5}\text{} \right) \end{aligned}$$

$$\begin{aligned} v_{y}=\frac{q\tau}{m}E_{y}-\frac{\tau qB}{m}v_{x}\#\left( \text{}\text{}\text{}\text{6}\text{} \right) \end{aligned}$$

Combining equations ($5$) and ($6$), we can get

$$\begin{aligned} \left( \begin{aligned} J_{x} \\ J_{y} \end{aligned} \right)=\frac{\sigma}{1+\left( \mu B \right)^{2}}\left( \begin{matrix} 1 & \mu B \\ -\mu B & 1 \end{matrix} \right)\left( \begin{aligned} E_{x} \\ E_{y} \end{aligned} \right)\#\left( \text{}\text{}\text{}\text{7}\text{} \right) \end{aligned}$$

where $\sigma=\frac{{nq}^{2}\tau}{m}$ is the electrical conductivity of the carrier.

Similar to the derivation of the single-band model above, we can get the two-band model:

$$\begin{aligned} \left( \begin{aligned} J_{x} \\ J_{y} \end{aligned} \right)=\left( \begin{matrix} \frac{\sigma_{1}}{1+\left( \mu_{1}B \right)^{2}}+\frac{\sigma_{2}}{1+\left( \mu_{2}B \right)^{2}} & \frac{\sigma_{1}\mu_{1}B}{1+\left( \mu_{1}B \right)^{2}}+\frac{\sigma_{2}\mu_{2}B}{1+\left( \mu_{2}B \right)^{2}} \\ -\frac{\sigma_{1}\mu_{1}B}{1+\left( \mu_{1}B \right)^{2}}-\frac{\sigma_{2}\mu_{2}B}{1+\left( \mu_{2}B \right)^{2}} & \frac{\sigma_{1}}{1+\left( \mu_{1}B \right)^{2}}+\frac{\sigma_{2}}{1+\left( \mu_{2}B \right)^{2}} \end{matrix} \right)\left( \begin{aligned} E_{x} \\ E_{y} \end{aligned} \right)\#\left( \text{}\text{}\text{}\text{8}\text{} \right) \end{aligned}$$

where $\sigma_{1}$ and $\sigma_{2}$ represent the electrical conductivities of carrier 1 and carrier 2, respectively, while $\mu_{1}$ and $\mu_{2}$ denote their corresponding mobilities. The carrier density of each carrier can be obtained by $\frac{\sigma_{1}}{q\mu_{1}}=n_{1}$ and $\frac{\sigma_{2}}{q\mu_{2}}=n_{2}$, which means a positive $n$ represents a hole-type carrier while a negative $n$ represents an electron-type carrier. Equation ($\text{8}$) has been widely applied to fit the electronic transport data in materials such as YBa_2_Cu_4_O_8_, Cd_3_As_2_, and black phosphorus [13-15]. Some studies have also utilized this formula to analyze the transport data of materials with anomalous Hall effects, like Co_3_Sn_2_S_2_ [16].

**The two-band model**. The motion of Bloch electrons on a certain band can be described by the following two equations:

$$\begin{aligned} \hbar\overset{\boldsymbol{\cdot}}{\boldsymbol{k}}=-q\boldsymbol{v}\times\boldsymbol{B}-q\boldsymbol{E}\#\left( \text{}\text{}\text{}\text{9}\text{} \right) \end{aligned}$$

$$\begin{aligned} \boldsymbol{v}=\frac{1}{\hbar}\nabla_{\boldsymbol{k}}E_{n}\left( \boldsymbol{k} \right)\boldsymbol{\#}\left( \text{}\text{}\text{}\text{10}\text{} \right) \end{aligned}$$

where $\boldsymbol{k}$ is the quasi-momentum of the Bloch electron, $\boldsymbol{v}$ is the velocity of the Bloch electron, $\boldsymbol{E}$ and $\boldsymbol{B}$ are the electric field and magnetic induction strength at the real space position where the electron is located, and $E_{n}\left( \boldsymbol{k} \right)$ is the eigenenergy level of the Bloch electron. Considering the Berry curvature, Equation (10) will have an additional term:

$$\begin{aligned} \boldsymbol{v}=\frac{1}{\hbar}\nabla_{\boldsymbol{k}}E_{n}\left( \boldsymbol{k} \right)+\frac{q}{\hbar}\boldsymbol{E}\times\boldsymbol{b}_{n}\left( \boldsymbol{k} \right)\boldsymbol{\#}\left( \text{}\text{}\text{}\text{11}\text{} \right) \end{aligned}$$

where $\boldsymbol{b}_{n}\left( \boldsymbol{k} \right)$ is the Berry curvature, which is defined as the curl of the Berry connection $\boldsymbol{A}^{n}\left( \boldsymbol{k} \right)$. For a two-dimensional system,

$$\begin{aligned} &\boldsymbol{A}^{n}\left( \boldsymbol{k} \right)=ⅈ\left\langle n\left( \boldsymbol{k} \right)\left| \frac{\partial}{\partial\boldsymbol{k}} \right|n\left( \boldsymbol{k} \right) \right\rangle\\ &=ⅈ\langle n(\boldsymbol{k})|(\frac{\partial n\left( \boldsymbol{k} \right)}{\partial k_{x}}{\overset{\boldsymbol{^}}{\boldsymbol{k}}}_{\boldsymbol{x}}+(\frac{\partial n\left( \boldsymbol{k} \right)}{\partial k_{y}}{\overset{\boldsymbol{^}}{\boldsymbol{k}}}_{\boldsymbol{y}})\rangle\\ &=ⅈ\langle n(\boldsymbol{k})|\frac{\partial n(\boldsymbol{k})}{\partial k_{x}}\rangle{\overset{\boldsymbol{^}}{\boldsymbol{k}}}_{\boldsymbol{x}}+ⅈ\langle n(\boldsymbol{k})|\frac{\partial n(\boldsymbol{k})}{\partial k_{y}}\rangle{\overset{\boldsymbol{^}}{\boldsymbol{k}}}_{\boldsymbol{y}}\#\left（ \text{}\text{}\text{}\text{12}\text{} \right） \end{aligned}$$

Thus, $\boldsymbol{b}_{n}\left( \boldsymbol{k} \right)=\nabla_{\boldsymbol{k}}\times\boldsymbol{A}^{n}\left( \boldsymbol{k} \right)$=0${\overset{^}{\boldsymbol{k}}}_{\boldsymbol{x}}+0{\overset{^}{\boldsymbol{k}}}_{\boldsymbol{y}}+\left( \frac{\partial A_{k_{y}}^{n}}{k_{x}}-\frac{\partial A_{k_{x}}^{n}}{k_{y}} \right){\overset{\boldsymbol{^}}{\boldsymbol{k}}}_{\boldsymbol{z}}\boldsymbol{=}\left( \frac{\partial A_{k_{y}}^{n}}{k_{x}}-\frac{\partial A_{k_{x}}^{n}}{k_{y}} \right){\overset{^}{\boldsymbol{k}}}_{\boldsymbol{z}}$, which means that for a two-dimensional system, the vector Berry curvature only has the component that is perpendicular to the system surface $b_{nz}\left( \boldsymbol{k} \right).$

Since $\boldsymbol{E}$ leads to a change in the momentum of the Bloch electron, Equation ($\text{11}$) can be written as

$$\begin{aligned} \boldsymbol{v}=\frac{1}{\hbar}\nabla_{k}E_{n}\left( \boldsymbol{k} \right)-\overset{\cdot}{\boldsymbol{k}}\times\boldsymbol{b}_{n}\left( \boldsymbol{k} \right)\boldsymbol{\#}\left( \text{}\text{}\text{}\text{13}\text{} \right) \end{aligned}$$

Where $\boldsymbol{v}_{\boldsymbol{a}}=-\overset{\cdot}{\boldsymbol{k}}\times\boldsymbol{b}_{n}\left( \boldsymbol{k} \right)$ is called the anomalous velocity. Correspondingly, the Hall current density contributed by this anomalous velocity would be

$$\begin{aligned} \boldsymbol{J}_{\boldsymbol{n}}=-\frac{e}{A}\sum_{n} \boldsymbol{v}_{\boldsymbol{a}}=\frac{-e}{A}\sum_{n} \frac{e}{\hbar}\boldsymbol{E}\times\boldsymbol{b}_{n}\left( \boldsymbol{k} \right) \\ &=\frac{e}{A}\sum_{n} \frac{e}{\hbar}\boldsymbol{b}_{n}\left( \boldsymbol{k} \right)\times\boldsymbol{E}=\overset{\boldsymbol{^}}{\boldsymbol{z}}\times\boldsymbol{E}\frac{e^{2}}{A\hbar}\sum_{n} b_{n}\left( \boldsymbol{k} \right)\#\left( \text{}\text{}\text{}\text{14}\text{} \right) \end{aligned}$$

where $n$ is the number of electrons on the corresponding band. If we define $\frac{e^{2}}{A\hbar}\sum_{\boldsymbol{m}} \boldsymbol{b}_{n}\left( \boldsymbol{k} \right)=\sigma_{xy}^{A}$, then we have

$$\begin{aligned} \boldsymbol{J}_{\boldsymbol{n}}=\sigma_{\mathrm{xy}}^{A}\overset{\boldsymbol{^}}{\boldsymbol{z}}\times\boldsymbol{E\#}\left( \text{}\text{}\text{}\text{15}\text{} \right) \end{aligned}$$

where $\overset{\boldsymbol{^}}{\boldsymbol{z}}$ is the unit vector pointing to the *z* direction (the direction perpendicular to the surface of the two-dimensional system). For two-dimensional ferromagnetic materials, the component of magnetization perpendicular to the sample surface $M_{z}$ is approximately proportional to $b_{nz}(\boldsymbol{k})$ (the proportionality coefficient may be positive or negative) ^11,12^. This implies that when an external magnetic field, applied perpendicular to the sample surface, is reversed—thereby flipping the direction of $M_{z}$—the sign of the anomalous Hall conductivity $\sigma_{xy}^{A}$ will also invert.

Since the anomalous Hall effect affects the transverse velocity of electrons, we can add the anomalous Hall conductivity $\sigma_{xy}^{A}$ into the off-diagonal elements of the electrical conductivity tensor in Equation to account for the anomalous Hall effect. This anomalous Hall conductivity-integrated model (the two-band model) is given as follows:

$$\begin{aligned} \left( \begin{aligned} J_{x} \\ J_{y} \end{aligned} \right)=\left( \begin{matrix} \frac{\sigma_{1}}{1+\left( \mu_{1}B \right)^{2}}+\frac{\sigma_{2}}{1+\left( \mu_{2}B \right)^{2}} & \frac{\sigma_{1}\mu_{1}B}{1+\left( \mu_{1}B \right)^{2}}+\frac{\sigma_{2}\mu_{2}B}{1+\left( \mu_{2}B \right)^{2}}+\sigma_{xy}^{A} \\ -\frac{\sigma_{1}\mu_{1}B}{1+\left( \mu_{1}B \right)^{2}}-\frac{\sigma_{2}\mu_{2}B}{1+\left( \mu_{2}B \right)^{2}}-\sigma_{xy}^{A} & \frac{\sigma_{1}}{1+\left( \mu_{1}B \right)^{2}}+\frac{\sigma_{2}}{1+\left( \mu_{2}B \right)^{2}} \end{matrix} \right)\left( \begin{aligned} E_{x} \\ E_{y} \end{aligned} \right)\#\left( \text{}\text{}\text{}\text{16}\text{} \right) \end{aligned}$$

Inverting the electrical conductivity tensor in Equation ($\text{16}$) would yield the expressions for both the MR $\rho_{xx}(B)$ and the Hall resistance $\rho_{xy}(B)$:

$$\begin{aligned} \rho_{xx}=\frac{\sigma_{1}+B^{2}{\mu_{2}}^{2}\sigma_{1}+\sigma_{2}+B^{2}{\mu_{1}}^{2}\sigma_{2}}{\left( 1+B^{2}{\mu_{2}}^{2} \right){\sigma_{1}}^{2}+2\sigma_{1}\left( \sigma_{2}+B^{2}\mu_{1}\mu_{2}\sigma_{2}+B\mu_{1}\left( 1+B^{2}{\mu_{2}}^{2} \right)\sigma_{xy}^{A} \right)+\left( 1+B^{2}{\mu_{1}}^{2} \right)\left( {\sigma_{2}}^{2}+2B\mu_{2}\sigma_{2}\sigma_{xy}^{A}+\left( 1+B^{2}{\mu_{2}}^{2} \right){\sigma_{xy}^{A}}^{2} \right)}\#\left（ \text{}\text{}\text{}\text{17}\text{} \right） \end{aligned}$$

$$\begin{aligned} \rho_{xy}=\frac{\sigma_{\mathrm{xy}}^{A}+B\left( \mu_{1}\left( \sigma_{1}+B^{2}{\mu_{2}}^{2}\sigma_{1} \right)+\mu_{2}\left( \sigma_{2}+B\mu_{2}\sigma_{xy}^{A} \right)+B{\mu_{1}}^{2}\left( \sigma_{xy}^{A}+B\mu_{2}\left( \sigma_{2}+B\mu_{2}\sigma_{xy}^{A} \right) \right) \right)}{\left( 1+B^{2}{\mu_{2}}^{2} \right){\sigma_{1}}^{2}+2\sigma_{1}\left( \sigma_{2}+B^{2}\mu_{1}\mu_{2}\sigma_{2}+B\mu_{1}\left( 1+B^{2}{\mu_{2}}^{2} \right)\sigma_{xy}^{A} \right)+\left( 1+B^{2}{\mu_{1}}^{2} \right)\left( {\sigma_{2}}^{2}+2B\mu_{2}\sigma_{2}\sigma_{xy}^{A}+\left( 1+B^{2}{\mu_{2}}^{2} \right){\sigma_{xy}^{A}}^{2} \right)}\#\left( \text{}\text{}\text{}\text{18}\text{} \right) \end{aligned}$$

In the two-band model, the five variables, $n_{1}$, $n_{2}$, $\sigma_{1}$, $\sigma_{2}$ and $\sigma_{xy}^{A}$, are independent in the sense that they are not constrained by a single functional relationship. The conductivity in each band obeys $\sigma_{i}=n_{i}e\mu_{i}$ ($i$ = 1, 2), where $e$ is the elementary charge, and $\mu$ is the carrier mobility. It can be seen that $\sigma_{i}$ depends on both carrier density $n_{i}$ and mobility $\mu_{i}$. Thus, for a given $n_{i}$, $\sigma_{i}$ can still vary via changes in $\mu_{i}$, and vice versa. Choosing ($n_{1}$, $n_{2}$, $\sigma_{1}$, $\sigma_{2}$ and $\sigma_{xy}^{A}$) as independent parameters is therefore equivalent to choosing ($n_{1}$, $n_{2}$, $\mu_{1}$, $\mu_{2}$ and $\sigma_{xy}^{A}$)—both represent five degrees of freedom in the model. Varying $\sigma_{i}$ at fixed $n_{i}$ corresponds physically to tuning the mobility, which is experimentally plausible. Hence, treating the five parameters as independently tunable parameters does not impose unphysical correlations among them.

Note that for the two-band model, the Hall angle $\theta$ satisfies $\tan\theta=\frac{E_{y}}{E_{x}}=\frac{\sigma_{1}\mu_{1}+\sigma_{2}\mu_{2}}{\sigma_{1}+\sigma_{2}}B= \frac{n_{2}\sigma_{1}^{2}+n_{1}\sigma_{2}^{2}}{en_{1}n_{2}\left( \sigma_{1}+\sigma_{2} \right)}B$, and the anomalous Hall angle $\nu$ satisfies $\tan\nu=\frac{\sigma_{xy}^{A}}{\sigma_{xx}}=\frac{\sigma_{xy}^{A}}{\frac{\sigma_{1}}{1+\left( \mu_{1}B \right)^{2}}+\frac{\sigma_{2}}{1+\left( \mu_{2}B \right)^{2}}}=\frac{\sigma_{xy}^{A}}{e^{2}\left( \frac{n_{1}^{2}\sigma_{1}}{\left( en_{1}+B\sigma_{1} \right)^{2}}+\frac{n_{2}^{2}\sigma_{2}}{\left( en_{2}+B\sigma_{2} \right)^{2}} \right)}$. These expressions show that the magnitudes of the Hall angle and the anomalous Hall angle are determined by the electronic parameters and serve as a direct parameter to influence our machine learning process.

**The modified model used for fitting.** According to the two-band model ($\text{16}$), the conductivity $\sigma_{xx}\left( B \right)=\frac{\sigma_{1}}{1+\left( \mu_{1}B \right)^{2}}+\frac{\sigma_{2}}{1+\left( \mu_{2}B \right)^{2}}$ should not exhibit hysteresis with the external magnetic field since any term that may reflect hysteresis is absent. For instance, $\sigma_{xy}^{A}$, which exhibits a sign change corresponding to magnetization direction reversal, can characterize hysteresis behavior and is absent from the expression for $\sigma_{xx}\left( B \right)$. Supplementary Figure 14a, b provides examples of the MR curve and the $\sigma_{xx}-B$ curve produced by the two-band model demonstrating that even though the MR curve can indeed exhibit hysteresis, the $\sigma_{xx}-B$ curve is symmetrical about the y-axis. However, it has been found experimentally that the $\sigma_{xx}-B$ curves also exhibit hysteresis. To accurately fit the experimental data, it is essential to account for the hysteresis observed in $\sigma_{xx}$. To address this, we introduced an additional phenomenological parameter $\alpha$ into the two-band model:

$$\begin{aligned} \left( \begin{aligned} J_{x} \\ J_{y} \end{aligned} \right)=\left( \begin{matrix} \frac{\sigma_{1}}{1+\left( \mu_{1}\left( B+\alpha\sigma_{xy}^{A} \right) \right)^{2}}+\frac{\sigma_{2}}{1+\left( \mu_{2}\left( B+\alpha\sigma_{xy}^{A} \right) \right)^{2}} & \frac{\sigma_{1}\mu_{1}B}{1+\left( \mu_{1}B \right)^{2}}+\frac{\sigma_{2}\mu_{2}B}{1+\left( \mu_{2}B \right)^{2}}+\sigma_{xy}^{A} \\ -\frac{\sigma_{1}\mu_{1}B}{1+\left( \mu_{1}B \right)^{2}}-\frac{\sigma_{2}\mu_{2}B}{1+\left( \mu_{2}B \right)^{2}}-\sigma_{xy}^{A} & \frac{\sigma_{1}}{1+\left( \mu_{1}\left( B+\alpha\sigma_{xy}^{A} \right) \right)^{2}}+\frac{\sigma_{2}}{1+\left( \mu_{2}\left( B+\alpha\sigma_{xy}^{A} \right) \right)^{2}} \end{matrix} \right)\left( \begin{aligned} E_{x} \\ E_{y} \end{aligned} \right)\#\left( \text{}\text{}\text{}\text{19}\text{} \right) \end{aligned}$$

In the remaining article, we refer to Equation ($19$) as the modified model. The intuition for adding $\alpha\sigma_{xy}^{A}$ to the denominators of the diagonal elements is that when the magnetization direction reverses, $\sigma_{xy}^{A}$ also changes sign, which makes the term $\alpha\sigma_{xy}^{A}$ shift the $\sigma_{xx}-B$ curve’s left half (starting from the maximum negative magnetic field to the positive coercive field) and right half (starting from the maximum positive magnetic field to the negative coercive field) move towards each other, thus characterizing the hysteresis behavior. Similarly, by inverting the tensor in Equation ($\text{19}$), we can get the expression for $\rho_{xx}$ and $\rho_{xy}$:

$$\begin{aligned} \rho_{xx}=\frac{\frac{\sigma_{1}}{1+{\mu_{1}}^{2}\left( B+\alpha\sigma_{\mathrm{xy}}^{A} \right)^{2}}+\frac{\sigma_{2}}{1+{\mu_{2}}^{2}\left( B+\alpha\sigma_{\mathrm{xy}}^{A} \right)^{2}}}{\left( \frac{\sigma_{1}}{1+{\mu_{1}}^{2}\left( B+\alpha\sigma_{\mathrm{xy}}^{A} \right)^{2}}+\frac{\sigma_{2}}{1+{\mu_{2}}^{2}\left( B+\alpha\sigma_{\mathrm{xy}}^{A} \right)^{2}} \right)^{2}+\frac{\left( \sigma_{\mathrm{xy}}^{A}+B\left( \mu_{1}\left( \sigma_{1}+B^{2}{\mu2}^{2}\sigma_{1} \right)+\mu_{2}\left( \sigma_{2}+B\mu_{2}\sigma_{\mathrm{xy}}^{A} \right)+B{\mu_{1}}^{2}\left( \sigma_{\mathrm{xy}}^{A}+B\mu_{2}\left( \sigma_{2}+B\mu_{2}\sigma_{\mathrm{xy}}^{A} \right) \right) \right) \right)^{2}}{\left( 1+B^{2}{\mu_{1}}^{2} \right)^{2}\left( 1+B^{2}{\mu_{2}}^{2} \right)^{2}}}\#\left( \text{}\text{}\text{}\text{20}\text{} \right) \end{aligned}$$

$$\begin{aligned} \rho_{xy}=\frac{\frac{B\mu_{1}\sigma_{1}}{1+B^{2}\mu-1^{2}}+\frac{B\mu_{2}\sigma_{2}}{1+B^{2}{\mu_{2}}^{2}}+\sigma_{\mathrm{xy}}^{A}}{\left( \frac{\sigma_{1}}{1+{\mu_{1}}^{2}\left( B+\alpha\sigma_{\mathrm{xy}}^{A} \right)^{2}}+\frac{\sigma_{2}}{1+{\mu_{2}}^{2}\left( B+\alpha\sigma_{\mathrm{xy}}^{A} \right)^{2}} \right)^{2}+\frac{\left( \sigma_{\mathrm{xy}}^{A}+B\left( \mu_{1}\left( \sigma_{1}+B^{2}{\mu_{2}}^{2}\sigma_{1} \right)+\mu_{2}\left( \sigma_{2}+B\mu_{2}\sigma_{\mathrm{xy}}^{A} \right)+B{\mu_{1}}^{2}\left( \sigma_{\mathrm{xy}}^{A}+B\mu_{2}\left( \sigma_{2}+B\mu_{2}\sigma_{\mathrm{xy}}^{A} \right) \right) \right) \right)^{2}}{\left( 1+B^{2}{\mu_{1}}^{2} \right)^{2}\left( 1+B^{2}{\mu_{2}}^{2} \right)^{2}}}\#\left( \text{}\text{}\text{}\text{21}\text{} \right) \end{aligned}$$

**Supplementary Figure** **14 |** $\boldsymbol{\rho}_{\boldsymbol{xx}}\boldsymbol{(B)}$ **and** $\boldsymbol{\sigma}_{\boldsymbol{xx}}\boldsymbol{(B)}$ **curves obtained by the two-band model.** (a) MR obtained from the two-band model exhibits hysteresis. (b) $\sigma_{xx}(B)$ generated by the two-band model does not exhibit hysteresis.

**Preprocessing experimental data.** Experimental electronic transport data often exhibit mixed signals, where $R_{xx}(B)$ measurements may contain contributions from $R_{xy}(B)$, and vice versa**.** This is because during electrode etching, it is difficult to ensure that the electrode ends are perfectly parallel along the current direction, nor can the transverse electrodes be made completely equal in width and parallel. To extract the true $R_{xx}(B)$ data, we apply a standard symmetrization procedure (Supplementary Fig. 15 a, b). Let $R_{xx}^{0}(B)$ denote the raw experimental data, which can be decomposed into two segments: $R_{xx(+\to-)}^{0}(B)$, which corresponds to $B$ sweeping from the maximum positive value to the negative maximum value, and $R_{xx(-\to+)}^{0}(B)$, which corresponds to $B$ sweeping from the maximum negative value to the positive maximum value. For the part where $B$ sweeps from the maximum positive value to the negative maximum value, the actual $R_{xx}(B)$ should be

$$\begin{aligned} R_{xx}\left( B \right)=\frac{R_{xx\left( +\to- \right)}^{0}\left( B \right)+R_{xx\left( -\to+ \right)}^{0}\left( -B \right)}{2}\#\left( 22 \right) \end{aligned}$$

For the part where $B$ sweeps from the maximum negative value to the positive maximum value, the actual $R_{xx}(B)$ should be

$$\begin{aligned} R_{xx}\left( B \right)=\frac{R_{xx\left( +\to- \right)}^{0}\left( B \right)+R_{xx\left( -\to+ \right)}^{0}\left( -B \right)}{2}\#\left( 23 \right) \end{aligned}$$

Similarly, to obtain the actual $R_{xy}(B)$ data, we need to perform an anti-symmetrization operation on the signals measured between the two electrodes that are perpendicular to the applied current direction (Supplementary Fig. 15 c, d). Assuming the experimental data is denoted as $R_{xy}^{0}(B)$, it should first be divided into two parts: $R_{xy(+\to-)}^{0}(B)$, which corresponds to $B$ sweeping from the maximum positive value to the negative maximum value, and $R_{xy(-\to+)}^{0}(B)$, which corresponds to $B$ sweeping from the maximum negative value to the positive maximum value. For the part where $B$ sweeps from the maximum positive value to the negative maximum value, the actual $R_{xy}(B)$ should be

$$\begin{aligned} R_{xy}\left( B \right)=\frac{R_{xx\left( +\to- \right)}^{0}\left( B \right)-R_{xx\left( -\to+ \right)}^{0}\left( -B \right)}{2}\#\left( 24 \right) \end{aligned}$$

For the part where $B$ sweeps from the maximum negative value to the positive maximum value, the actual $R_{xy}(B)$ should be

$$\begin{aligned} R_{xy}\left( B \right)=\frac{R_{xx\left( -\to+ \right)}^{0}\left( B \right)-R_{xx\left( +\to- \right)}^{0}\left( -B \right)}{2}\#\left( 25 \right) \end{aligned}$$

It is important to note that for a two-dimensional system, $R_{xy}$ equals to $\rho_{xy}$, while $R_{xx}=\rho_{xx}\frac{L}{w}$, where $L$ and $w$ represent the sample's length (the distance between the two electrodes along the current direction) and width (the distance between the two electrodes perpendicular to the current direction), respectively.

**Curve fitting for data collected in Fe_5_GeTe_2_ samples.** We fitted experimental MR curves obtained from two Fe_5_GeTe_2_ samples at different temperatures and gate voltages to the modified model (Equation ($\text{20}$)). Prior to fitting, the $\rho_{xx}$ data is divided into two segments: the left segment comprises data from the negative maximum magnetic field to the positive coercive field, while the right segment includes data from the positive maximum field to the negative coercive field. During the fitting process, Equation ($\text{20}$) is used to fit the left segment, while for the right segment, the formula obtained by changing $\sigma_{xy}^{A}$ in Equation ($\text{20}$) to -$\sigma_{xy}^{A}$ is used for fitting. Both segments are fitted using the same set of parameters. Supplementary Figures 16 and 17 show the fitting curves of the two Fe_5_GeTe_2_ samples. It can be seen that the modified model can achieve excellent fitting of the experimental data.

**Supplementary Figure** **15.** Symmetrization and anti-symmetrization of raw data obtained from experiments. (a) Example of raw magnetoresistance $R_{xx}^{0} (\Omega)$ obtained experimentally. (b) $R_{xx} (\Omega)$ obtained by symmetrizing the data shown in a**.** (c) Example of raw Hall resistance $R_{xy}^{0} (\Omega)$ obtained experimentally. (d) $R_{xy} (\Omega)$ obtained by anti-symmetrizing the data shown in c.

**Supplementary Figure** **16.** MR data and fitted $\rho_{xx}-B$ curves under different temperatures and gate voltages obtained from Fe_5_GeTe_2_ sample Ⅰ. In each $\rho_{xx}-B$ graph corresponding to a specific temperature and gate voltage, the gray points denote the original data, while the red and blue curves represent the fitted curves for the left and right segments of the data, respectively. Different background colors represent different curve types, as indicated by the legends at the bottom.

**Supplementary Figure** **17.** MR data and fitted $\rho_{xx}-B$ curves under different temperatures and gate voltages obtained from Fe_5_GeTe_2_ sample Ⅱ. In each $\rho_{xx}-B$ graph corresponding to a specific temperature and gate voltage, the gray points denote the original data, while the red and blue curves represent the fitted curves for the left and right segments of the data, respectively. Different background colors represent different curve types, as indicated by the legends at the bottom.

8. **Comparison between the revised model and the empirical relation**

For $\rho_{xy}$ data, a widely used method for quantitative analysis to fit the data is to utilize the empirical relation [17,18]

$$\begin{aligned} \rho_{xy}=R_{H}B+R_{\mathrm{AHE}}\#\left( \text{}\text{}\text{}\text{26}\text{} \right) \end{aligned}$$

where $R_{H}$ is the normal Hall effect coefficient, and $R_{\mathrm{AHE}}$ is called the anomalous Hall resistance, which is usually determined by the material. It has been nearly a century since this empirical relation was proposed, and it has become a common method for fitting $\rho_{xy}$ data. When the anomalous Hall effect is weak, $\frac{1}{R_{H}}$ exhibits an approximate proportionality to the total carrier concentration, making $R_{H}$ a useful parameter for estimating both the carrier type and density. For ferromagnetic materials, $R_{\mathrm{AHE}}\approx R_{S}M$, where $M$ is the magnetization, and $R_{S}$ is a proportionality coefficient called the anomalous Hall coefficient.

By performing a Taylor expansion on Equation ($\text{18}$), we obtain

$$\begin{aligned} \rho_{xy}=\frac{\sigma_{xy}^{A}}{\sigma_{1}^{2}+2\sigma_{1}\sigma_{2}+\sigma_{2}^{2}+{\sigma_{xy}^{A}}^{2}}+\frac{\left( \mu_{1}\sigma_{1}+\mu_{2}\sigma_{2} \right)\left( \sigma_{1}^{2}+2\sigma_{1}\sigma_{2}+\sigma_{2}^{2}-{\sigma_{xy}^{A}}^{2} \right)B}{\left( \sigma_{1}^{2}+2\sigma_{1}\sigma_{2}+\sigma_{2}^{2}+{\sigma_{xy}^{A}}^{2} \right)^{2}}+{O(B}^{2})\#\left( \text{}\text{}\text{}\text{27}\text{} \right) \end{aligned}$$

For the first-order term of $B$, by expanding it as a Taylor series for $\sigma_{xy}^{A}$ around zero, we obtain

$$\begin{aligned} \frac{\left( \mu_{1}\sigma_{1}+\mu_{2}\sigma_{2} \right)\left( \sigma_{1}^{2}+2\sigma_{1}\sigma_{2}+\sigma_{2}^{2}-{\sigma_{xy}^{A}}^{2} \right)}{\left( \sigma_{1}^{2}+2\sigma_{1}\sigma_{2}+\sigma_{2}^{2}+{\sigma_{xy}^{A}}^{2} \right)^{2}} \\ &=\frac{\mu_{1}\sigma_{1}+\mu_{2}\sigma_{2}}{\left( \sigma_{1}+\sigma_{2} \right)^{2}}-\frac{3\left( \mu_{1}\sigma_{1}+\mu_{2}\sigma_{2} \right){\sigma_{xy}^{A}}^{2}}{\left( \sigma_{1}+\sigma_{2} \right)^{4}}+O{(\sigma_{xy}^{A})}^{4}\#\left（ \text{}\text{}\text{}\text{28}\text{} \right） \end{aligned}$$

We can see that when $\sigma_{xy}^{A}\ll\sigma_{1}+\sigma_{2}$, the coefficient of the first-order term of $B$ is

$$\begin{aligned} \frac{\mu_{1}\sigma_{1}+\mu_{2}\sigma_{2}}{\left( \sigma_{1}+\sigma_{2} \right)^{2}}=\frac{\frac{\sigma_{1}}{n_{1}q}\sigma_{1}+\frac{\sigma_{2}}{n_{2}q}\sigma_{2}}{\left( \sigma_{1}+\sigma_{2} \right)^{2}}=\frac{\frac{\sigma_{1}^{2}}{n_{1}q}+\frac{\sigma_{2}^{2}}{n_{2}q}}{\left( \sigma_{1}+\sigma_{2} \right)^{2}}\#\left( \text{}\text{}\text{}\text{29}\text{} \right) \end{aligned}$$

By comparing Equations ($\text{29}$) and ($\text{26}$), we can see that

$$\begin{aligned} R_{H}=\frac{\frac{\sigma_{1}^{2}}{n_{1}q}+\frac{\sigma_{2}^{2}}{n_{2}q}}{\left( \sigma_{1}+\sigma_{2} \right)^{2}}\#\left( \text{}\text{}\text{}\text{30}\text{} \right) \end{aligned}$$

We would arrive at Equation ($\text{30}$) again if we apply the same analysis to the modified model with Eq. $\text{21}$). It can be seen from Equation ($\text{30}$) that $R_{H}$ is no longer simply the reciprocal of the sum of carrier concentrations, nor is it the sum of the reciprocals of carrier concentrations. Instead, it is the sum of the reciprocals of carrier concentrations, each weighted by a factor related to conductivity.

Supplementary Figure 18 shows fitting results for two sets of *bowtie* data. Supplementary Figure 18a and b show the $\rho_{xx}(B)$ and $\rho_{xy}(B)$ data and their fit curves at $T$= 75 K and $V_{g}$ = 1.5 V. One can see that both $\rho_{xx}(B)$ and $\rho_{xy}(B)$ can be well fitted using the same set of parameters. Supplementary Figure 18c further demonstrates the fitting result of $\rho_{xy}(B)$ using the empirical relation with Equation ($\text{26}$). The carrier concentrations obtained by fitting using the revised model are $n_{1}=-1.467\times{10}^{11} \mathrm{cm}^{-2}$ and $n_{2}=1.012\times{10}^{15}\mathrm{cm}^{-2}$, indicating a hole-type carrier. While the fitting result using the empirical relation yields $R_{H}=-1.146\times{10}^{-2}\Omega/T$, which means that the carrier concentration is $n_{\mathrm{eff}}=\frac{1}{R_{H} q}=-8.726\times{10}^{16}\mathrm{cm}^{-2}$, indicating an electron-type carrier. As can be seen from the fitting results, $\sigma_{xy}^{A}$ is about several orders of magnitude smaller than $\sigma_{1}+\sigma_{2}$, roughly satisfying the condition of $\sigma_{\mathrm{xy}}^{A}$<<$\sigma_{1}+\sigma_{2}$. When calculating the carrier concentration using Equation ($\text{30}$), we obtain $n_{\mathrm{cal}}=-3.509\times{10}^{15}\mathrm{cm}^{-2}$, which indeed returns to the conclusion that the majority carrier is electron as predicted by the empirical formula. Therefore, the modified model can reveal more detailed carrier information that cannot be shown by the empirical relation.

Supplementary Figure 18d and e shows the original data and fitting results for $\rho_{xx}(B)$ and $\rho_{xy}(B)$ obtained at $T$ = 50 K and $V_{g}$ = 1.9 V. Supplementary Figure 18f further demonstrates the fitting result of $\rho_{xy}(B)$ using the empirical relation. Similar to the previous case, analysis by the modified model suggest that hole is the majority carrier, while analysis by the empirical relation indicates that electron is the majority carrier with a concentration of $n_{\mathrm{eff}}=-4.785\times{10}^{16}$ cm^-2^. When calculated using Equation ($\text{30}$), we obtain $n_{\mathrm{cal}}=-4.366\times{10}^{15}$ cm^-2^, again returning to the conclusion of the empirical formula. While simultaneous fitting of both $\rho_{xx}(B)$ and $\rho_{xy}(B)$ data using the same set of parameters would be ideal, practical limitations have been encountered. For the data shown in Supplementary Fig. 16, simultaneous fitting of both $\rho_{xx}(B)$ and $\rho_{xy}(B)$ data for sample Ⅰ was largely successful, with the exception of *double-hump-spike* curves, for which fitting was performed only on $\rho_{xx}(B)$. For the data shown in Supplementary Fig. 17, fitting was performed only on $\rho_{xx}(B)$.

**Supplementary Figure** **18.** Curve fitting for MR using the modified two-band model, and for anomalous Hall resistance using both the modified two-band model and the empirical formula. (a) MR data and the MR curves fitted using the modified two-band model for the Fe_5_GeTe_2_ sample I at $T$ = 75 K and $V_{g}$ = 1.5 V. The fitting parameters are displayed on the right side of the graph. (b) $\rho_{xy}(B)$ data and the $\rho_{xy}-B$ curves fitted using the two-band model for the Fe_5_GeTe_2_ sample I at $T$ = 75 K and $V_{g}$ = 1.5 V. The fitting parameters are the same as in a. (c) $\rho_{xy}(B)$ data and the $\rho_{xy}-B$ curve fitted using the empirical formula for the Fe_5_GeTe_2_ sample Ⅰ at $T$ = 75 K and $V_{g}$ = 1.5 V. The fitting parameters are shown on the right side of the graph. (d) MR data and the MR curves fitted using the two-band model for the Fe_5_GeTe_2_ sample I at $T$ = 50 K and $V_{g}$ = 1.9 V. The fitting parameters are displayed on the right side of the graph. (e) $\rho_{xy}(B)$ data and the $\rho_{xy}-B$ curves fitted using the two-band model for the Fe_5_GeTe_2_ sample I at $T$ = 50 K and $V_{g}$ = 1.9 V. The fitting parameters are the same as in a. (f) $\rho_{xy}(B)$ data and the $\rho_{xy}-B$ curve fitted using the empirical formula for the Fe_5_GeTe_2_ sample Ⅰ at $T$ = 50 K and $V_{g}$ = 1.9 V. The fitting parameters are shown on the right side of the graph.

9. **Transport measurement for gated Fe_5_GeTe_2_ devices**

**Device fabrication.** The Fe_5_GeTe_2_ single crystals were grown by the chemical vapor transport method. The Hall-bar electrodes were patterned onto the substrate through standard electron-beam lithography and metal evaporation procedures. The Fe_5_GeTe_2_ flakes were exfoliated onto PDMS (polydimethylsiloxane) and transferred onto prepatterned substrates. Then, they were immediately spin-coated with PMMA (polymethyl methacrylate) for protection from air. All exfoliation and transfer processes were performed inside a N_2_ gas glovebox (oxygen and water levels below 0.1 ppm).

**Electronic transport measurement.** Electronic transport measurements were carried out in an Oxford Instruments TeslatronPT system equipped with a 12 T superconducting magnet. longitudinal and Hall voltages were detected with synchronized lock-in amplifiers (SR830, Stanford Research Systems). The gate voltage was supplied by a direct current source meter (Keithley 2400). the devices were directly zero-field cooled from 300 K to 1.5 K with a cooling rate of 1 K/min. The gating electrolyte is a solution of LiClO_4_ and polyethylene oxide (PEO), where a mixed powder of LiClO_4_ and PEO (0.1 and 0.33 g) was dissolved in 5 ml anhydrous methanol [19,20]. We stirred the electrolyte for over 24 hours and kept it at 50 ℃ before use. The sample, after being covered with the electrolyte solution and subsequently annealed under vacuum at 100 ℃ for 0.5 hours to evaporate methanol, was transferred into a vacuum chamber for transport measurements. Within this chamber, the devices underwent in situ annealing at 370 K for 30 minutes to remove residual organic solvent from the electrolyte. Following this, the gate voltage was applied at 330 K before cooling down to 1.5 K. Magnetic measurements were then conducted during a warm-up process.

10. **Two-band model analysis for MR data of gated devices**

Supplementary Figure 19 presents the carrier densities of Fe_5_GeTe_2_ (Sample I) measured at varying temperatures and gate voltages. Below a gate voltage of 1.9 V, the system hosts both hole and electron carriers, with hole being the majority carrier. Notably, the hole density displays a non-monotonic temperature dependence, initially decreasing before rising again, while the electron density follows an opposite trend—first increasing and then decreasing. The inflection point for the hole density occurs at approximately 125 K, a temperature at which prior studies [20] have reported a change of the magnetic easy axis direction occurs. Given that itinerant electrons near the Fermi level are known to modulate magnetic moments in this material [21-23], they likely play a key role in governing the observed magnetic anisotropy. The coupling between itinerant electrons and localized magnetic moments remains a complex problem; however, our modified model demonstrates that the shift in the magnetic easy axis direction correlates with changes in carrier density. This approach offers a novel tool for probing and manipulating magnetic anisotropy in such systems.

Supplementary Figure 19 further reveals that under high gate voltages ($V_{g}$ ≥ 2.2V), a transition in the AH-MR type is typically accompanied by a pronounced change in carrier density. For instance, at $V_{g}$= 2.4 V and 2.5 V with $T$ ≤ 100 K, both carriers remain electron-type. However, at $T$ = 100 K, one electron-type carrier converts to a hole-type, while the density of the other electron-type carrier decreases. This transition coincides with the emergence of a *double*-*hump* AH-MR type at 100 K. When the gate voltage is smaller than 2.2 V, at 100 K the AH-MR type changes from *butterfly* at lower temperatures to *upward-plateau* at higher temperatures. This occurrence coincides with one electron-type carrier exhibiting a rapid increase in density, while the density of the other carrier experiences a rapid decrease. Supplementary Figure 20a,b shows the carrier densities for Fe_5_GeTe_2_ (sample Ⅱ) under varying gate voltages. One can see that at different gate voltages and temperatures, the major carriers are all hole-type carriers. Notably, a distinct turning point in the majority carrier density occurs within the temperature range of approximately 125–140 K. Supplementary Figure 20a further shows that with increasing gate voltage, the density of the majority carriers generally decreases, demonstrating the role of gate voltage in modulating carrier concentration.

Supplementary Figure 20c presents the carrier mobility versus carrier density relationship for Fe_5_GeTe_2_ (sample I), revealing an inverse correlation between these parameters. This observed trend of decreasing mobility with increasing carrier density is consistent with numerous previous studies on semiconductors [24-27]. The underlying physical mechanism can be attributed to enhanced carrier-carrier and carrier-phonon scattering at higher carrier densities^24^. A similar mobility-density relationship is observed for Fe_5_GeTe_2_ sample Ⅱ (Supplementary Figure 20d), where mobility likewise decreases with increasing density. Supplementary Figure 21 shows the temperature dependence of the anomalous Hall conductivity $\sigma_{xy}^{A}$ under different gate voltages for Fe_5_GeTe_2_ sample Ⅰ and Ⅱ, respectively. One can see from Supplementary Fig. 21a that at low gate voltages ($V_{g}\leq$1.9V), the absolute value of $\sigma_{xy}^{A}$ decreases with increasing temperature, whereas at high gate voltages ($V_{g}>$1.9V), ${|\sigma}_{xy}^{A}|$ first exhibits a slight increase before decreasing as temperature rises. Additionally, ${|\sigma}_{xy}^{A}|$ decreases with increasing gate voltage. Similar trends are clearly observed in Supplementary Fig. 21b. Since ${|\sigma}_{xy}^{A}|$ reflects the strength of the anomalous Hall effect, these results demonstrate that both gate voltage and temperature can effectively modulate the anomalous Hall effect.

**Supplementary Figure** **19.** Carrier density as a function of temperature under different gate voltages for Fe_5_GeTe_2_ sample Ⅰ. From a to f**,** the gate voltages are 0, 1.5, 1.9, 2.2, 2.4 and 2.5 V. In each panel, the red line shows the density of the first carrier and corresponds to the left *y*-axis, while the blue line shows the density of the second carrier and corresponds to the right *y*-axis.

**Supplementary Figure** **20.** Electronic transport parameters obtained from Fe_5_GeTe_2_ sample Ⅰ and Ⅱ by fitting experimental data to the modified model. (a, b) Carrier density as a function of temperature at different voltages for the main carrier in sample Ⅱ. (c, d) Carrier mobility as a function of carrier density at different gate voltages for sample Ⅰ and Ⅱ.

**Supplementary Figure** **21.** Anomalous Hall conductivities obtained from Fe_5_GeTe_2_ sample Ⅰ and Ⅱ. (a, b) Anomalous Hall conductivity as a function of temperature at different gate voltages for sample Ⅰ and Ⅱ, respectively.

11. **Machine learning prediction of GMR**

To provide a systematic analysis of the conditions conducive to giant magnetoresistance (GMR), we present phase diagrams in Supplementary Fig. 23, generated using the same electronic parameters as in Fig. 3 of the main text. The increase of MR at *B* = 16 T with respect to MR at *B* = 0 T is given as $r_{\mathrm{GMR}}=\frac{\rho_{xx}(B = 16 T)-\rho_{xx}(B = 0 T)}{\rho_{xx}(B = 0 T)}$. Three important observations need to be addressed here. First, GMR is most likely to emerge when at least one carrier type is an electron with positive anomalous Hall conductivity. Second, the carrier density required for GMR increases with higher electrical conductivity. Third, when the carrier with higher conductivity also exhibits a higher density, the $r_{\mathrm{GMR}}$ value increases, making the GMR effect more pronounced. These insights underscore the critical role of optimizing carrier properties—particularly electrical conductivity and carrier density—to engineer more pronounced GMR phenomena.

**Supplementary Figure 22.** Phase diagrams highlighting LMR regions. (a) Magnetoresistance increase in the LMR region, defined as $r_{\mathrm{LMR}}=\frac{\rho_{xx}(16 T)-\rho_{xx}(0)}{\rho_{xx}(0)}$, plotted as a function of carrier density in a ferromagnetic material with $\sigma_{xy}^{A}\neq0$. The specific conductivity values are as follows: $\sigma_{1}=0.1$S, $\sigma_{2}=0.095$S, $\sigma_{xy}^{A}=0.08$ S. The inset displays a magnified view of a specific region. The deep blue and light blue colors both signify regions where LMR does not occur, with deep (or light) blue indicating the density of hole-type carriers larger (or smaller) than the density of electron-type carriers. (b) MR curves along the path shown in the inset in a. Here $n_{2}$ is set to be $1\times{10}^{12}$ cm^-2^. (c) $r_{\mathrm{GMR}}$ as a function of carrier densities in a non-magnetic material with $\sigma_{xy}^{A}=0$. The specific conductivity values are as follows: $\sigma_{1}=0.1$S, $\sigma_{2}=0.095$S, $\sigma_{xy}^{A}=0$S. The inset displays a magnified view of a specific region. (d) MR curves along the path shown in the inset in **c**. Here $n_{1}$ is set to be equal to $n_{2}$.

**Supplementary Figure** **23.** Phase diagrams highlighting GMR regions. (a-d) Phase diagrams of $r_{\mathrm{GMR}}$ with varying carrier densities, where $\sigma_{2}=0.95\sigma_{1}$. From b to e, $\sigma_{1}$ equals to 1 S, 0.1 S, 0.01 S and 0.001 S, respectively. In every diagram, $\sigma_{xy}^{A}$ remains at 0.08$\sigma_{1}$. (e-f) Phase diagrams of $r_{\mathrm{GMR}}$ with varying carrier densities, where $\sigma_{2}=0.1\sigma_{1}$. From f to i,$\sigma_{1}$ equals to 1 S, 0.1 S, 0.01 S and 0.001 S, respectively.

12. **Machine learning prediction of QAHE-like magnetoresistance**

Supplementary Figure 24a shows a phase diagram of $\rho_{xx}^{H_{C}}$ as a function of carrier densities, highlighting the parameter space region where QAHE-like AH-MR curves emerge. Note that the increasing trend of $\rho_{xx}^{H_{C}}$ with decreasing carrier density (increased mobility) is consistent with theoretical predictions that systems with higher mobility are more likely to display quantized Hall plateaus. Supplementary Figure 24b shows the temperature-dependent AH-MR data obtained experimentally from a typical topological insulator Bi_2_Se_3_. The observed increase in peak value $\rho_{xx}^{H_{C}}$ with decreasing temperature can be attributed to the reduced electron scattering and consequent enhancement of carrier mobility, showing the capability of our phase diagram to predict the general trends in QAHE. We further provide phase diagrams with carrier density ranging from $1\times{10}^{12}$ cm^-2^ to$1\times{10}^{13}$ cm^-2^ under two different electrical conductivity combinations in Supplementary Fig. 24c, d. Note that the two-band model failed to provide a good fit of the experimental data shown in Supplementary Fig. 24b. We attribute this limitation to the absence of topological invariants in the model. Nevertheless, as demonstrated above, the two-band model remains capable of predicting the general trend of the peak feature in QAHE and could offer valuable insights into searching for QAHE from the perspective of electronic parameters. While the two-band model fails to quantitatively reproduce the experimental AH-MR in QAHE systems, the Chalker–Coddington–style network model is capable of doing so and represents a promising direction for future research [28,29].

**Supplementary Figure** **24.** MR in the QAHE-like region as a function of carrier densities. (a) MR in the QAHE-like region as a function of carrier densities when $\sigma_{1}=1 \mathrm{mS}$, $\sigma_{xy}^{A}=0.08 mS$,$\sigma_{2}=0.95\mathrm{mS}$. $\rho_{xx}^{H_{C}}$ represents the peak value. (b) Experimental QAHE data obtained from (Cr, V)_y_(Bi_1-x_Sb_x_)_2-y_Te_3_ at different temperatures. (c) Phase diagram obtained when $\sigma_{1}=1 S$,$\sigma_{2}=0.95 S$ and $\sigma_{xy}^{A}=0.08 S$. (d) Phase diagram obtained when $\sigma_{1}=1 S$,$\sigma_{2}=0.1 S$ and $\sigma_{xy}^{A}=0.08 S$.

13. **AH-MR phase diagrams with varying** $\boldsymbol{\sigma}_{\boldsymbol{xx}}$ **and** $\boldsymbol{\sigma}_{\boldsymbol{xy}}$

It is well known that the anomalous Hall effect can be attributed to the intrinsic or extrinsic mechanism based on the power law relation between $\sigma_{xx}$ and $\sigma_{xy}$ [30]. The expressions for $\sigma_{xx}$ and $\sigma_{xy}$ according to Equation ($\text{16}$) are

$$\begin{aligned} \sigma_{xx}=\frac{\sigma_{1}}{1+\left( \mu_{1}B \right)^{2}}+\frac{\sigma_{2}}{1+\left( \mu_{2}B \right)^{2}}\#\left( 31 \right) \end{aligned}$$

$$\begin{aligned} \sigma_{xy}=\frac{\sigma_{1}\mu_{1}B}{1+\left( \mu_{1}B \right)^{2}}+\frac{\sigma_{2}\mu_{2}B}{1+\left( \mu_{2}B \right)^{2}}+\sigma_{xy}^{A}\#\left( 32 \right) \end{aligned}$$

Note that when $B$ equals to 0, $\sigma_{xy}=\sigma_{xy}^{A}$ and $\sigma_{xx}=\sigma_{1}+\sigma_{2}$. We set $B=0$ and calculated $\sigma_{xx}$ and $\sigma_{xy}$ values for different combinations of the five electronic parameters. Specifically, we set the conductivity of one carrier type to 10^-6^ S, while varying the conductivity of the other carrier from 10^-6^ S to 10^2^ S. Regarding $\sigma_{xy}$, we varied $\sigma_{xy}^{A}$ from 10^-12^ S to 10^3^ S. Based on these parameters, we obtained AH-MR phase diagrams shown in Extended Data Fig. 2 in the main text. To facilitate a direct comparison between our AH-MR phase diagrams and the $\sigma_{xy}-\sigma_{xx}$ relationship established by Nagaosa et al.^28^, we fixed the system thickness at 1 μm and converted the units to S/cm. It should also be addressed that $\mu=\frac{q\tau}{m}$, where the effective mass $m$ and relaxation time $\tau$ are both positive, and the sign of $q$—which is also the sign of $\mu$—indicates whether the carrier is electron or hole. A positive (negative) sign indicates hole- (electron-) type carrier. Supplementary Figure 25 provides such AH-MR phase diagrams under different carrier mobility values. Interestingly, the regions with large anomalous Hall angle show distinct negative magnetoresistance (*bowtie* and *downward-plateau*). Note that the expression for MR in the absence of $\sigma_{xy}^{A}$, as obtained by inverting the electrical conductivity tensor in Equation ($\text{8}$), always has a positive derivative and will not exhibit negative MR:

$$\begin{aligned} \frac{d\rho_{xx}}{dB}=\frac{2B\left( \mu_{1}-\mu_{2} \right)^{2}\sigma_{1}\sigma_{2}\left( \sigma_{1}+\sigma_{2} \right)}{\left( \left( 1+B^{2}{\mu_{2}}^{2} \right){\sigma_{1}}^{2}+\left( 1+B^{2}{\mu_{1}}^{2} \right){\sigma_{2}}^{2}+2\sigma_{1}\left( \sigma_{2}+B^{2}\mu_{1}\mu_{2}\sigma_{2} \right) \right)^{2}}>0\# \end{aligned}$$

However, the derivative of $\rho_{xx}$ according to Equation ($\text{17}$) is

$$\frac{d\rho_{xx}}{dB}=(2B\left( \mu_{1}-\mu_{2} \right)^{2}\sigma_{1}\sigma_{2}(\sigma_{1}+\sigma_{2})-2(\mu_{1}\left( \sigma_{1}+B^{2}{\mu2}^{2}\sigma_{1} \right)^{2}+(\mu_{1}+\mu_{2})(1+B\mu_{1}+B(-1+B\mu_{1})\mu_{2})(1+B(\mu_{2}+\mu_{1}(-1+B\mu_{2})))\sigma_{1}\sigma_{2}+\mu_{2}\left( \sigma_{2}+B^{2}{\mu_{1}}^{2}\sigma_{2} \right)^{2})\sigma_{xy}^{A}-2B(\left( \mu_{1}+B^{2}\mu_{1}{\mu_{2}}^{2} \right)^{2}\sigma_{1}+\left( \mu_{2}+B^{2}{\mu_{1}}^{2}\mu_{2} \right)^{2}\sigma_{2}){\sigma_{xy}^{A}}^{2})/{((1+B^{2}{\mu_{2}}^{2}){\sigma_{1}}^{2}+2\sigma_{1}(\sigma_{2}+B^{2}\mu_{1}\mu_{2}\sigma_{2}+B\mu_{1}(1+B^{2}{\mu_{2}}^{2})\sigma_{xy}^{A})+(1+B^{2}{\mu_{1}}^{2})({\sigma_{2}}^{2}+2B\mu_{2}\sigma_{2}\sigma_{xy}^{A}+(1+B^{2}{\mu_{2}}^{2}){\sigma_{xy}^{A}}^{2}))}^{2}$$

, which can be negative. Supplementary Figure 26 displays the difference (*Δ*) between the slope of the boundary line and the Nagaosa line. Notably, *Δ* increases as the carrier mobility of the major carrier decreases, and remains unchanged with varying carrier mobility of the minor carrier.

**Supplementary Figure** **25.** AH-MR phase diagrams illustrating AH-MR types as a function of $\sigma_{xx}$ and $\sigma_{xy}$. The phase diagrams are obtained under different mobility values, as shown by the horizontal axis and the vertical axis.

**Supplementary Figure** **26.** Slope difference between the large anomalous Hall angle region boundary line and the Nagaosa line. (a) The phase diagram as a function of $\sigma_{xx}$ and $\sigma_{xy}$ obtained when $\mu_{1}=-1\times{10}^{4}$ (cm^2^/(V·s)) and $\mu_{2}=1\times{10}^{4}$(cm^2^/(V·s)). The yellow dots are the data points separating the region displaying the *downward-plateau* AH-MR type and the region displaying the *bowtie* AH-MR type. The red line is the fitted line of these yellow points. (b) The phase diagram as a function of $\sigma_{xx}$ and $\sigma_{xy}$ obtained when $\mu_{1}=-1\times{10}^{4}$(cm^2^/(V·s)) and $\mu_{2}=1\times{10}^{4}$(cm^2^/(V·s)), the red dashed line is the same line as the red line in a, and the orange dashed line is the fitted line of the Nagaosa line in the extrinsic regime. (c) *Δ*, which is the difference between the slope of the boundary line and the Nagaosa line, as a function of the carrier mobilities.

14. **Possible physical origin of different AH-MR states**

Based on the correspondence between the Nagaosa line and our phase diagrams (Fig. 6), we provide analysis on the transition of AH-MR states with large correlation coefficients (Fig. 4a) and give the following possible mechanisms responsible for these transitions.

**Dirty metal to clean metal transition (*bowtie* to *butterfly*):** As shown in Fig. 6e and f, the bowtie state appears primarily in the dirty metal regime, while the butterfly state dominates in moderately dirty and superclean regions. This indicates that the bowtie-to-butterfly transition is driven by a decrease in disorder—a shift from dirty to cleaner metallic behavior.

**Dirty metal to moderately dirty metal transition** **(*bowtie* to *downward-plateau*):** As shown in Fig. 6e, the *bowtie* state appears primarily in the dirty metal region, whereas the *downward-plateau* state is more common in the moderately dirty metal region. This suggests that the transition from *bowtie* to *downward-plateau* is likely due to a shift from the dirty metal regime to the intrinsic mechanism regime.

**Dirty metal to superclean metal transition (*downward-plateau* to the *butterfly*)**: As shown in Fig. 6e, the transition from the *downward-plateau* to the *butterfly* state likely reflects a shift from intrinsic to extrinsic dominant mechanisms for the anomalous Hall effect, as the butterfly state occupies a larger superclean metal region.

**Enhanced anomalous velocity (*upward-butterfly* to *butterfly*):** As shown in Fig. 6e and f, the *upward-butterfly* state is characterized by a classical $B^{2}$ dependence at low fields, signifying Lorentz-force-induced magnetoresistance. In contrast, the *butterfly* state again appears in intrinsic and extrinsic regimes. This suggests the transition to the *butterfly* state is likely induced by an increase in the intrinsic anomalous velocity contribution.

**Increased conductivity of the minor carrier (*valley-spike* to *butterfly*):** Figures 6c and d show that both these states occur within the dirty metal region, implying a more subtle transition mechanism that requires further investigations. However, Fig. 4e indicates that the transition between them is strongly linked to a change in electrical conductivity of the minor carrier, suggesting this parameter may be the primary driving force.

**Mobility enhancement (*bowtie* to *flat-spike*):** There is a notable trend that the region occupied by the *flat-spike* state at higher mobilities ($\mu={10}^{6}\left( \mathrm{cm}^{2}/\left( V\cdot s \right) \right)$ and ${10}^{7}\left( \mathrm{cm}^{2}/\left( V\cdot s \right) \right)$) is largely occupied by the bowtie state at lower mobility ($\mu={10}^{5}\left( \mathrm{cm}^{2}/\left( V\cdot s \right) \right)$. This indicates that the transition from *bowtie* to *flat-spike* is likely due to an increase in mobility.

**References**

1.	Samuel AL. Some studies in machine learning using the game of checkers. *IBM Journal of Research and Development* 1959; **3**: 210–29.

2.	Epstein R, Roberts G, Beber G eds. *Parsing the Turing Test: Philosophical and Methodological Issues in the Quest for the Thinking Computer*. Dordrecht: Springer Netherlands, 2009.

3.	Fukunaga K, Hostetler L. The estimation of the gradient of a density function, with applications in pattern recognition. *IEEE Transactions on Information Theory* 1975; **21**: 32–40.

4.	Lee M, Onose Y, Tokura Y *et al.* Hidden constant in the anomalous Hall effect of high-purity magnet MnSi. *Phys Rev B* 2007; **75**: 172403.

5.	Miyasato T, Abe N, Fujii T *et al.* Crossover behavior of the anomalous Hall effect and anomalous Nernst effect in itinerant ferromagnets. *Phys Rev Lett* 2007; **99**: 086602.

6.	Potashnik SJ, Ku KC, Chun SH *et al.* Effects of annealing time on defect-controlled ferromagnetism in Ga_1-x_Mn_x_As. *Applied Physics Letters* 2001; **79**: 1495–7.

7.	Leighton C, Terry I, Becla P. Metallic conductivity near the metal-insulator transition in Cd_1-x_MnxTe. *Phys Rev B* 1998; **58**: 9773–82.

8.	Shestakov AV, Fazlizhanov II, Yatsyk IV *et al.* Investigations of magnetic properties Hg_0.865_Mn_0.135_Te by the electron spin resonance method. *IEEE Magnetics Letters* 2020; **11**: 1–5.

9.	Klambauer G, Unterthiner T, Mayr A *et al.* Self-normalizing neural networks. *Advances in Neural Information Processing Systems*. Vol 30. Curran Associates, Inc., 2017.

10.	Berry MV. Quantal phase factors accompanying adiabatic changes. *Proc R Soc A* 1997; **392**: 45–57.

11.	Xiao D, Chang M-C, Niu Q. Berry phase effects on electronic properties. *Rev Mod Phys* 2010; **82**: 1959–2007.

12.	Jungwirth T, Niu Q, MacDonald AH. Anomalous Hall effect in ferromagnetic semiconductors. *Phys Rev Lett* 2002; **88**: 207208.

13.	Rourke PMC, Bangura AF, Proust C *et al.* Fermi-surface reconstruction and two-carrier model for the Hall effect in YBa_2_Cu_4_O_8_. *Phys Rev B* 2010; **82**: 020514.

14.	Li C-Z, Li J-G, Wang L-X *et al.* Two-carrier transport induced Hall anomaly and large tunable magnetoresistance in Dirac semimetal Cd_3_As_2_ nanoplates. *ACS Nano* 2016; **10**: 6020–8.

15.	Akiba K, Miyake A, Akahama Y *et al.* Two-carrier analyses of the transport properties of black phosphorus under pressure. *Phys Rev B* 2017; **95**: 115126.

16.	Yang S-Y, Noky J, Gayles J *et al.* Field-modulated anomalous Hall conductivity and planar Hall effect in Co_3_Sn_2_S_2_ nanoflakes. *Nano Lett* 2020; **20**: 7860–7.

17.	Smith AW, Sears RW. The Hall effect in permalloy. *Phys Rev* 1929; **34**: 1466–73.

18.	Pugh EM. Hall effect and the magnetic properties of some ferromagnetic materials. *Phys Rev* 1930; **36**: 1503–11.

19.	Deng Y, Yu Y, Song Y *et al.* Gate-tunable room-temperature ferromagnetism in two-dimensional Fe_3_GeTe_2_. *Nature* 2018; **563**: 94–9.

20.	Tang M, Huang J, Qin F *et al.* Continuous manipulation of magnetic anisotropy in a van der Waals ferromagnet via electrical gating. *Nat Electron* 2023; **6**: 28–36.

21.	Yamamoto SJ, Si Q. Metallic ferromagnetism in the Kondo lattice. *Proc Natl Acad Sci* 2010; **107**: 15704–7.

22.	Egger R, Schoeller H. RKKY interaction and Kondo screening cloud for strongly correlated electrons. *Phys Rev B* 1996; **54**: 16337–40.

23.	Andreani LC, Pavarini E, Liviotti E *et al.* Competition between Kondo effect and magnetic interaction in Kondo systems. *Physica B: Condensed Matter* 1997; **230–232**: 523–8.

24.	Farvacque J-L, Bougrioua Z. Carrier mobility versus carrier density in Al_x_Ga_1-x_N/GaN quantum wells. *Phys Rev B* 2003; **68**: 035335.

25.	Thurber WR, Mattis RL, Liu YM *et al.* Resistivity‐dopant density relationship for phosphorus‐doped silicon. *J Electrochem Soc* 1980; **127**: 1807.

26.	Masetti G, Severi M, Solmi S. Modeling of carrier mobility against carrier concentration in arsenic-, phosphorus-, and boron-doped silicon. *IEEE Trans Electron Devices* 1983; **30**: 764–9.

27.	Masetti G, Solmi S. Relationship between carrier mobility and electron concentration in silicon heavily doped with phosphorus. *IEE Journal on Solid-State and Electron Devices* 1979; **3**: 65–8.

28.	Chalker JT, Coddington PD. Percolation, quantum tunnelling and the integer Hall effect. *J Phys C: Solid State Phys* 1988;**21**:2665.

29.	Lee D-H, Wang Z, Kivelson S. Quantum percolation and plateau transitions in the quantum Hall effect. *Phys Rev Lett* 1993; **70**: 4130–3.

30.	Nagaosa N, Sinova J, Onoda S *et al.* Anomalous Hall effect. *Rev Mod Phys* 2010; **82**: 1539–92.
